# From Synthesis to Therapeutics: Bioactive‐Coated Zein Nanoparticles in Drug Delivery

**DOI:** 10.1002/bip.70088

**Published:** 2026-03-15

**Authors:** Milena Ferreira de Lima, Andy Joel Taipe Huisa, Azael da Silva Neto, Clarice Beatriz Gonçalves Silva, Iago Dillion Lima Cavalcanti, Karolliny Barbosa de Araújo, Mariane Cajubá de Britto Lira‐Nogueira, Nereide Stela Santos Magalhães, Priscila Gubert

**Affiliations:** ^1^ Keizo Asami Institute, iLIKA, Federal University of Pernambuco Recife Brazil; ^2^ Graduate Program in Biology Applied to Health (PPGBAS), Federal University of Pernambuco Recife Brazil; ^3^ Institute of Biological Sciences (ICB), Physiological Sciences Post Graduation Program, Federal University of Rio Grande – FURG Rio Grande Brazil; ^4^ Postgraduate Program in Pharmaceutical Sciences (PPGCF ‐ UFPE), Center for Quality Control of Medicines and Related Products (NCQMC), Federal University of Pernambuco Recife Brazil; ^5^ Laboratory of Nanotechnology Biotechnology and Cell Culture, Academic Center of Vitória, Federal University of Pernambuco (CAV/UFPE) Recife Brazil; ^6^ Laboratory of Pharmaceutical Nanotechnology (LNFarm) Federal University of Pernambuco Recife Brazil

**Keywords:** coating materials, drug delivery systems, nanotechnology, therapeutic

## Abstract

Polymeric zein nanoparticles (ZNP), derived from corn protein, are biodegradable drug carriers with high stability and low synthesis costs. Their amphiphilic nature allows efficient encapsulation of both hydrophilic and lipophilic drugs, making them promising for drug delivery. However, their instability under physiological pH can limit therapeutic efficacy, necessitating protective coatings for improved absorption. This review discusses synthesis methods, coating materials, and biological activities of bioactive‐coated ZNP. We found that the antisolvent method is the most commonly used due to its simplicity and cost‐effectiveness, while chitosan is the preferred coating material. ZNP exhibit antioxidant, anticancer, anesthetic, antidiabetic, hypoglycemic, and immunogenic properties, as demonstrated in both in vitro and in vivo studies. Their ability to enhance bioavailability, reduce toxicity, and enable targeted drug delivery highlights their potential in nanomedicine.

## Graphical abstract

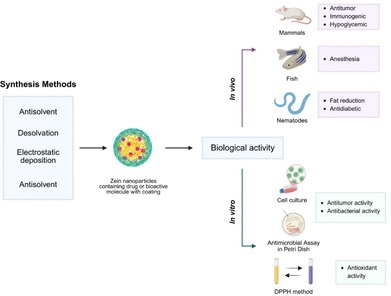

## 1 Introduction

In the pharmaceutical industry, nanoscience has made significant strides in improving and optimizing drug delivery methods, diagnostics, imaging, biosensors, and drug release mechanisms. The unique properties of nanomaterials make it possible to improve bioavailability of drugs, increase permeability through biological barriers, and protect against degradation when encapsulated [1, 2, 3]. Among the various nanocarrier options, including inorganic nanoparticles, dendrimers, liposomes, nanocrystals, and carbon nanotubes, polymeric nanoparticles have received considerable attention in scientific research [4].

**FIGURE 1:**
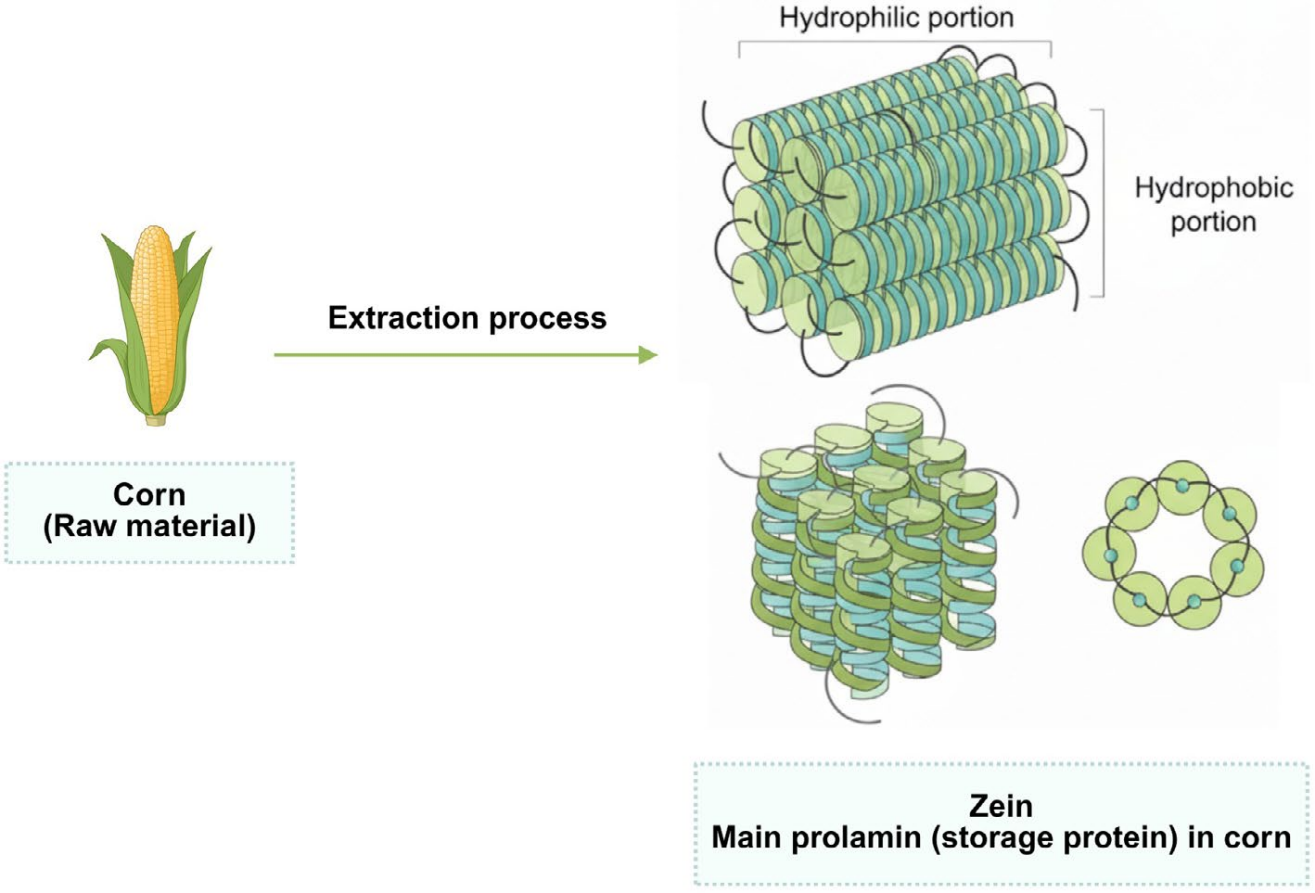
Illustration of the zein structure, showing its hydrophobic and hydrophilic regions, characterizing it as an amphiphilic molecule.

The use of protein‐based polymers, particularly biodegradable ones, offers several advantages. These include greater stability for volatile pharmaceutical agents, cost‐effectiveness, and ease of large‐scale production, resulting in higher concentrations of drugs at specific sites—making them ideal candidates for therapies, vaccines, contraceptives, antibiotics, and more specific treatments due to their ability to modulate the release and bioavailability of drugs [5, 6]. One of these polymers used in synthesis is zein, an amphiphilic protein found in corn kernels (Figure 1). Zein, despite its inherent hydrophobicity, has the ability to self‐assemble into various structures, such as microspheres, films, fibers, nanoparticles, and composites.

Plant proteins beyond corn zein have also emerged as viable materials for polymeric nanoparticle systems. Proteins such as soy isolate, pea protein, wheat gliadin, kafirin from sorghum, and rice bran protein can assemble into stable nanostructures with distinct surface characteristics and release behaviors. These botanical polymers differ considerably in hydrophobicity, amino‐acid composition, and structural organization, which ultimately shapes their stability, loading capacity, and interactions within biological environments [7, 8, 9]. Even so, zein remains the most widely adopted option due to its extensive characterization, low production cost, and broad availability, supporting both research use and scalable manufacturing.

Zein‐based drug delivery systems allow the sustained release of drugs without the need for chemical crosslinkers. However, it is important to note that pure zein‐based systems tend to aggregate at neutral or physiological pH levels, lack membrane permeability, are not specific to the administration site, and do not show precise drug release profiles. To meet these challenges, the researchers managed to coat the zein nanoparticles with different materials ([10, 11]).

**FIGURE 2:**
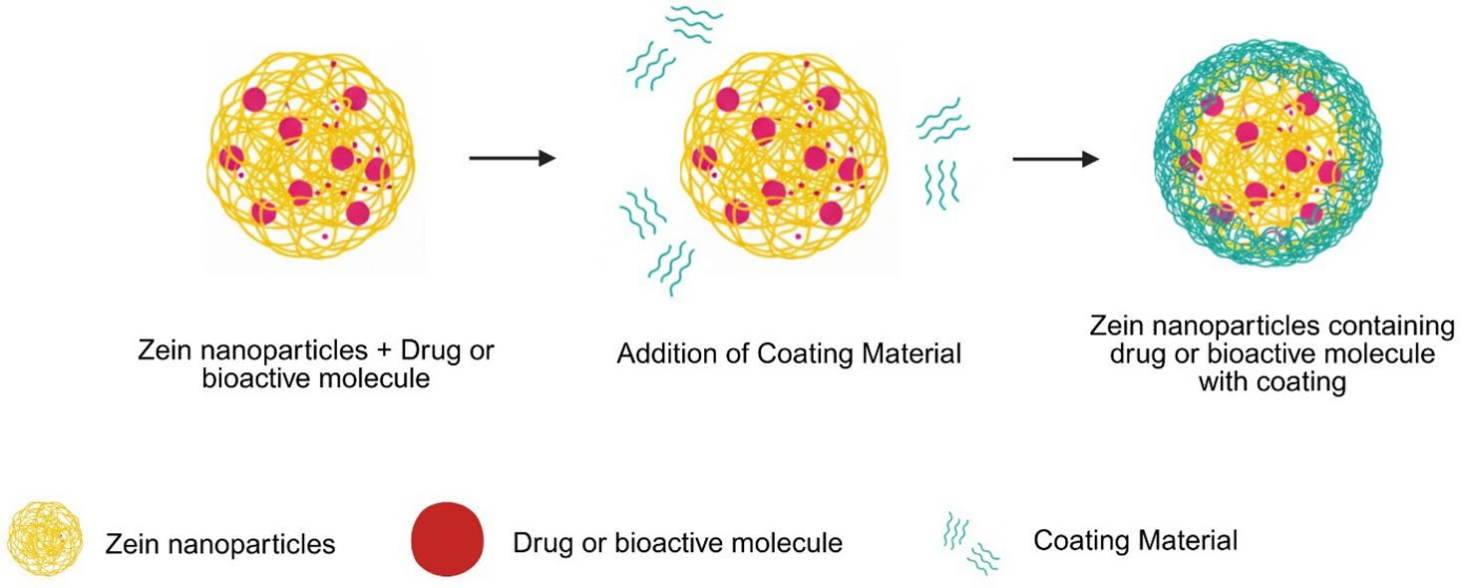
Illustration of zein nanoparticles carrying bioactives with and without coating.

As shown in Figure 2, zein nanoparticles carrying drugs can be coated to improve their stability and enhance their performance in the body. In this way, coated zein nanoparticles (ZNPs) have attracted attention due to their efficiency in encapsulating bioactive compounds. Not only do they improve parameters such as solubility and distribution, but they also reduce potential toxic effects through prolonged release, protecting them from environmental factors such as humidity and heat. In addition, zein is classified as a non‐toxic and biodegradable polymer and is generally recognized as safe (GRAS) [12, 13].

This review brings together current knowledge on coated zein nanoparticles (ZNPs), highlighting the main synthesis techniques, commonly applied coating materials, and significant findings from both in vitro and in vivo studies with relevance to healthcare applications. By connecting these elements, it offers a clear picture of prevailing research trends and helps inform future experimental strategies.

While several recent reviews discuss the general synthesis of ZNPs, few focus on coated systems or examine how surface modifications influence biological behavior. Comprehensive analyses linking coating approaches to in vitro and in vivo outcomes remain limited. Here, we address this gap by presenting current methods for preparing coated ZNPs and summarizing their biological evaluation, providing a practical framework to guide ongoing and future research.

## 2 Scope and Approach

This review highlights recent advancements in zein‐based delivery systems, focusing on the synergistic effects of co‐encapsulated bioactive compounds, improved stability, bioavailability, and controlled release mechanisms. The integration of zein with other biopolymers for hybrid systems is also discussed. A comprehensive literature review was carried out across four scientific databases using the search terms “nanoparticles,” “zein,” “coated,” and “drug.” This search identified 125 papers in SCOPUS, 212 in Web of Science (WOS), and 101 in PubMed, all published between 2012 and October 2025, with no restrictions regarding country or language. For the purposes of this review, we included original research articles and review articles were excluded.

**FIGURE 3:**
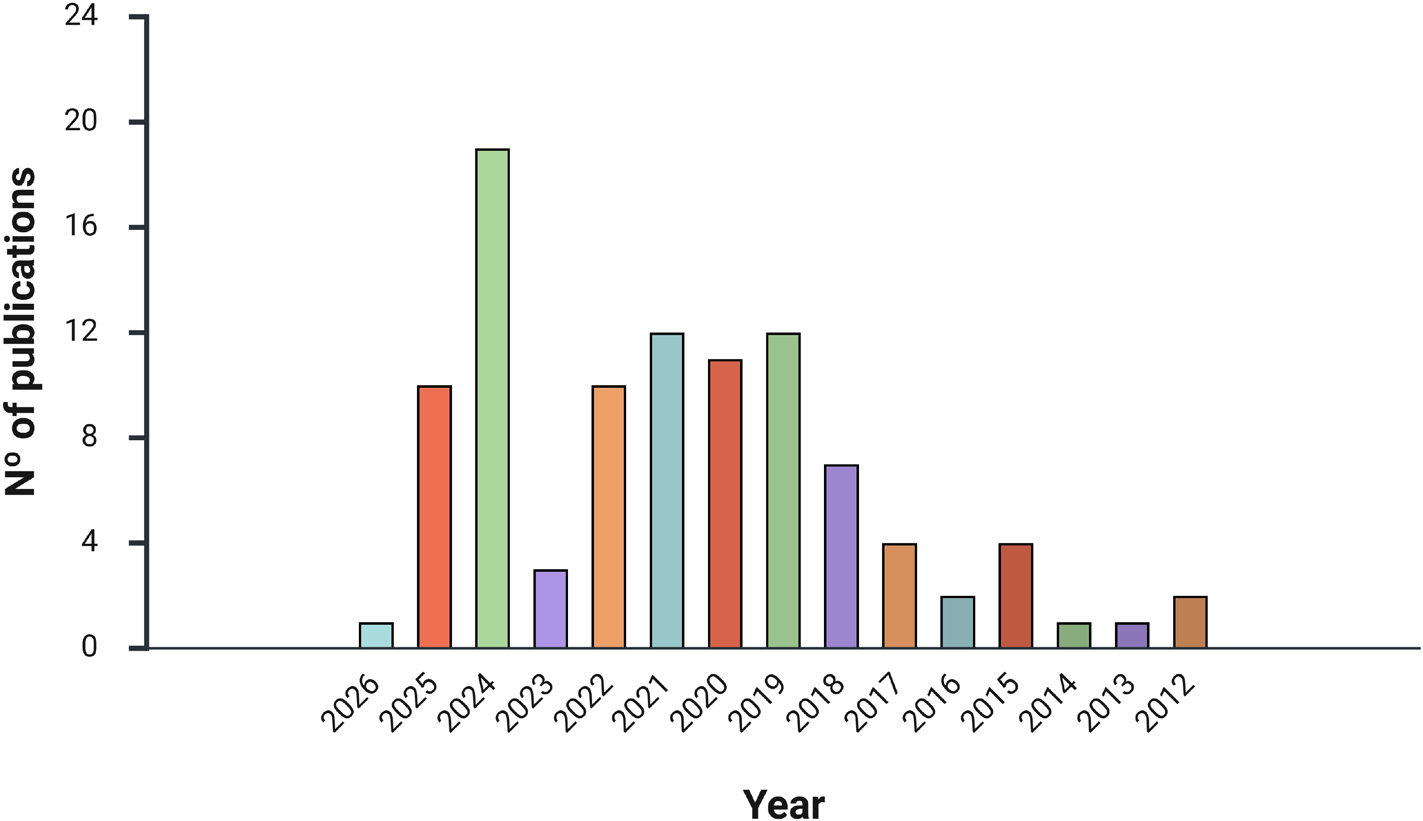
Chronology of publication of publications on coated zein nanoparticles selected with the descriptors “nanoparticles,” “zein,” “coated,” and “drug.”

After removing duplicate records and screening studies based on the predefined inclusion criteria, a total of 95 articles were deemed eligible for inclusion in this review. Notably, the number of articles on this topic showed a significant increase, especially from 2019 onward, as illustrated in Figure 3. Some reasons for the increase in publications include the notable advancements in technology and methodology, as well as the growing interest in sustainable materials.

The criteria for inclusion involved research articles that either developed zein nanoparticles with coatings, demonstrating their potential applications in the field of healthcare, testing these coated ZNPs in in vivo or in vitro models. Excluded from the review were articles falling into categories such as review articles, book chapters, encyclopedias, conference abstracts, mini‐reviews, short communications, and those that deviated from the core theme of the study. Original articles were included, with detailed methodology and results, as well as a broad discussion of the data obtained.

## 3 Synthesis Methods of Coated Zein Nanoparticles

The synthesis of nanoparticles is determined by several key factors, such as the type of drug to be loaded, the solvent employed, the coating material, and the intended application. Some production methods may leave behind solvent residues, which can introduce toxicity in both in vitro and in vivo experiments. Therefore, selecting a synthesis strategy that aligns with the intended system is essential, taking into account the drug, the coating, and the final purpose of the nanoparticles.

Several methods are currently employed to produce ZNPs prior to coating, differing in the type of drug carried, the synthesis technique, the coating material, and their potential applications, as summarized in Table 1. Among these techniques, they collectively account for approximately 70% of all selected articles, with the antisolvent approach being the most frequently cited (58.16%), followed by desolvation (8.16%) and electrostatic deposition (4.08%). Accordingly, they are described in detail in the following sections.

**TABLE 1:** Synthesis method, drug carried, and coating material.

Synthesis method	Drug	Coating	Particle size (nm)	Encapsulation efficacy (%)	References
Antisolvent	Benznidazole	Eudragit L100‐55	209	36–60	Pilicita et al. [14]
Antisolvent	Cinnamaldehyde and naringin	Lactoferrin and sodium caseinate	268.5	73.61–79.54	Mohamed et al. [15]
Antisolvent	Colchicine	Eudragit S100	104.3	59.8	Taymouri et al. [16]
Antisolvent	Curcumin	Dextran sulphate	180	96	Albogamy et al. [17]
Antisolvent	Curcumin	Alginate oligosaccharides	116.5	89.7	Jiang et al. [18]
Antisolvent	Curcumin	Tremella polysaccharides	164.0–225.2	93.34	Li et al. [19]
Antisolvent	Curcumin	Dextran sulfate	135	85.37	Yuan et al. [20]
Antisolvent	Curcumin	Xanthan gum	179	60–80	Zhang et al. [21, 22, 23]
Antisolvent	Curcumin	Ethanol‐soluble polysaccharide	253–266	88.7%–80.4%	Zhang et al. [24]
Antisolvent	Curcumin	Silk sericin	330–400	80–90	Zhu et al. [25]
Antisolvent	Curcumin	Carboxylic curdlans	183	66.7	Yu et al. [26]
Antisolvent	curcumin	Carboxymethyl cellulose	277.5	70	Öztekin et al. [27]
Antisolvent	Curcumin	Chitosan and genipin	185–190	82.3	Sha et al. [28]
Antisolvent	Curcumin	Gum Arabic	192.5–216	87–92.4	Ye et al. [29]
Antisolvent	Curcumin and piperine	Chitosan, hialuronic acid	599	90.4	Chen et al. [30]
Antisolvent	Docetaxel and cynomorium songaricum polysaccharide	Green tea polyphenols/iron coordination complex	274	45.0	Yu et al. [31]
Antisolvent	Eugenol	Chitosan	100	—	Ferreira et al. [32]
Antisolvent	Fucoxanthin	Chitosan; soybean polysaccharide	230	91	Zhao et al. [33]
Antisolvent	Gambogenic acid	Polydopamine	312	82.18	Zha et al. [34]
Antisolvent	Guavinoside B	Caseinate	176.2–178.7	84.11–88.70	Yang et al. [35]
Antisolvent	Honokiol	Polysialic acid	107.2 nm	79.2	Zhang et al. [36]
Antisolvent	Hypericin	Polietilenoglicol (PEG)/caseinates	92.7 ± 3.0	57.8	Abdelsalam et al. [37]
Antisolvent	Hyperoside	Pectin	272–298 nm	33.2–90.5	Wang et al. [38]
Antisolvent	Magnolol	Chondroitin sulfate	142.27–164.36 nm	65.34–80.71	Wang et al. [39]
Antisolvent	Naringin	Glutamine (Gln)‐modified chito‐oligosaccharides	116	88.25	Guo et al. [40]
Antisolvent	Niclosamide	Bovine serum albumin	200	—	Rejinold et al. [41]
Antisolvent	OMV‐F4 e OMV‐F18	Gantrez AN–mannosamine polymer	220–280	—	Matías et al. [42]
Antisolvent	Only zein	Chitosan	181	—	Lima et al. [43]
Antisolvent	Proanthocyanidins	Pectin	404.35	—	Li et al. [44]
Antisolvent	Propolis	Carboxymethyl chitosan	197–220	83	Zhang et al. [45]
Antisolvent	Quercetin	Polysaccharides	200	82.5	Li et al. [46]
Antisolvent	Quercetin	Fucoidan	211.7	80.54–90.83	Zhang et al. [21, 22, 23]
Antisolvent	Quercetin	Sodium caseinate and chitosan	188	82.78	Zhou et al. [47]
Antisolvent	Quercetin	Trimethylated chitosan	200–250	90	Dai et al. [48]
Antisolvent	Quercetin	Gelatin and carboxymethyl starch	189–219.1	95.2	Zhang et al. [49]
Antisolvent	Quercetin	Alginate‐pectin	298.6–475.8	86	Wan et al. [50]
Antisolvent	Quercetin	Chitosan	199.50–225.47	59.36–74.95	Liu et al. [51, 52]
Antisolvent	Resveratrol	Caseinate–dextran conjugates	224	—	Davidov‐Pardo et al. [53]
Antisolvent	Tannic acid	Pectin	166	89	Liang et al. [54]
Antisolvent	Tannic acid and resveratrol	Pectin	166.8	51.5–77.2	Liang et al. [55]
Antisolvent	Tanshinone	Pectin	132	79.41	Elmizadeh et al. [56]
Antisolvent	Thymol	Caseinates	200	—	Kang‐Kang et al. [57]
Antisolvent	β‐caroteno	Carboxymethyl chitosan	243.9	92.7	Wang et al. [58]
Antisolvent co‐precipitation	Curcumin	Ethylcellulose	167	63	Hasankhan et al. [59]
Antisolvent co‐precipitation	Insulin	Carboxy‐ methylated short‐chain amylose	168	90.5	Ji et al. [60]
Antisolvent co‐precipitation	Insulin	Chitosan	311.32	89.6	Ji et al. [61]
Antisolvent nanoprecipitation	Ellipticine	Sodium caseinate and polyethylene imine complex	137.6–166.7	94.8	Pourhossein et al. [62]
Antisolvent precipitation and electrophoretic deposition	Tannic acid	Copper‐doped bioactive glasses and sodium carboxymethyl cellulose	213–255	99	Hadzhieva et al. [63]
Antisolvent precipitation and electrostatic deposition	Fusidic acid	Lactoferrin	80.78	60.5	Marey et al. [64]
Antisolvent with modifications	Curcumin	Oxidized dextran	150	90	Rodriguez et al. [65]
Atomizing/antisolvent precipitation	Docetaxel	Soy lecithin and carboxymethyl chitosan	206.9	79.22	Wu et al. [66]
Co‐extrusion method	Paclitaxel	B16 cancer cell membrane	95.4–289.99	—	Huang et al. [67]
Complex coacervation	Doxorubicin hydrochloride	Complex of metal–tannic acid	156.4–181.7	91	Liang et al. [68]
Desolvation	Insulin	Gantrez AN‐thiamine conjugate	258–345	—	Inchaurraga et al. [69]
Desolvation	Insulin	Poly(anhydride)‐thiamine conjugate	250	80	Inchaurraga et al. [70]
Desolvation	Insulin	Gantrez AN‐PEG conjugate	248	88.7	Martínez‐López et al. [71]
Desolvation	Insulin	Poly(ethylene glycol)	263	84.8	Reboredo et al. [72]
Desolvation	Only zein	Poly(ethylene glycol)	200	—	Reboredo et al. [73]
Desolvation	Only zein	Poly(ethylene glycol) 35,000	200	—	Reboredo et al. [10]
Desolvation/deslocation	*Escherichia coli*	Gantrez–mannosamine conjugate	211	70	Berzosa et al. [74]
Electrostatic deposition	Curcumin	Alginate; carrageenan; pectin; gum arabic; carboxymethyl cellulose	160–210	80	Chang et al. [75]
Electrostatic deposition	Curcumin	Pectin	112–201.5	61.6–91.7	Chang et al. [76]
Electrostatic deposition	Lutein	Sophorolipid	200	90	Yuan et al. [77]
Electrostatic deposition	Zein/caseinate complex	Pectin	200	—	Chang et al. [78]
Emulsification‐solvent evaporation	Carbamazepine	Chitosan	221.46	62.28	Alak et al. [79]
Ethanol‐injection	Atorvastatin	lecithin	191.95	70	Elgendy et al. [80]
Ionic complexation	Doxorubicin	Tannic acid/FeII; tannic acid/AlIII; tannic acid/EuIII	135.9–145.9	78.4–83.1	Liang et al. [81]
Ionic complexation	Doxorubicin	(PEG)/tannin acid (TA) complex	221.3–275.7	81.8–88.6	Liang et al. [82]
Liquid antisolvent precipitation	Resveratrol	Maillard conjugates of sodium caseinate and dextran	180–198	83%	Davidov‐Pardo et al. [83]
Liquid–liquid dispersion	Curcumin	Sodium caseinate and Sodium alginate complex	185.63–191.10	36.10–76.06	Liu et al. [84]
Liquid–liquid dispersion	Resveratrol	Chitosan	295	51	Pauluk et al. [85]
Liquid–liquid dispersion	Thymol	Caseinates	398.90–521.80	80.51–82.89	Zhang et al. [86]
Liquid–liquid phase separation	indole‐3‐carbinol (I) or 3,30‐diindolylmethane (D)	Chitosan	113.5–89.1	77.79–78.08	Luo et al. [87]
Liquid–liquid phase separation	Simvastatin	Caseinate	131	89	Ahmed et al. [88]
Low‐energy phase separation	Curcumin	Water‐soluble chitosan	66–170	94.9	Liang et al. [89]
Low‐energy phase separation	Vitamin D3	Carboxymethyl chitosan	200	87.9	Luo et al. [90]
Nanoprecipitation	—	Sodium deoxycholate	100–200	—	Gagliardi et al. [91]
Nanoprecipitation	Genistein	Tretinoin	154.5	—	Kamel et al. [92]
Nanoprecipitation	Outer membrane vesicles (OMV) obtained from *Escherichia coli*	Gantrez‐mannosamine polymer conjugate	132	—	Matías et al. [93]
Nanoprecipitation	Paclitaxel	Sodium deoxycholate	103–158	40	Gagliardi et al. [94]
Nanoprecipitation	Resveratrol	Hydrophilic pectin and Eudragit S 100	100–200	94	Contado et al. [95]
Nanoprecipitation	Ceftazidime and tobramycin	Chitosan	315–335	55	Campos et al. [96]
New method	Folic acid and caffeic acid	Chitosan	176.3	CA: 15.1–64.1|FA: 5.9–84.1	Wusigale et al. [97]
Oxidative self‐polymerization	Doxorubicin	Tannic acid	155.8	68.6	Liang et al. [98]
pH‐driven	curcumin	Shellac	≅30	96	Lv et al. [99]
pH‐driven	Curcumin	Quaternary ammonium chitosan	218.2	89.3	Liu et al. [51, 52]
pH‐driven	Quercetin	Auricularia cornea Ehrenb polysaccharides	185.6	75.8	Wang et al. [100]
Phase separation	Exemestane and luteolin	PEGylated phospholipids, lactoferrin	229.5–500.9	61.8–88.3	El‐Lakany et al. [101]
Phase separation	Resveratrol	Caseinates	100	81.42	Shaomin et al. [102]
Phase separation	Curcumin	Polydopamine	53.2–150	36–79	Zhang et al. [22]
Phase separation	Resveratrol	Chitosan	94–132	91	Khan et al. [103]
Sacrificial template	Astaxanthin	Chitosan‐α‐lipoic acid copolymer and sodium alginate	239.07	88.13	Wang et al. [104]
Sacrificial template	Quercetin/doxorubicin	Chitosan	218.8	67.32	Da Paz Do Nascimento et al. [105]
Thin‐film hydration	Terbinafine	Dextran sulphate	273.2	9.2%–93.1%	Al‐Sawahli et al. [106]
Ultrasonic‐antisolvent method	Curcumin	Carboxymethylated corn fiber gum	158.17 to 380.93	91.19	Ma et al. [107]

### 3.1 Antisolvent/Nanoprecipitation

**FIGURE 4:**
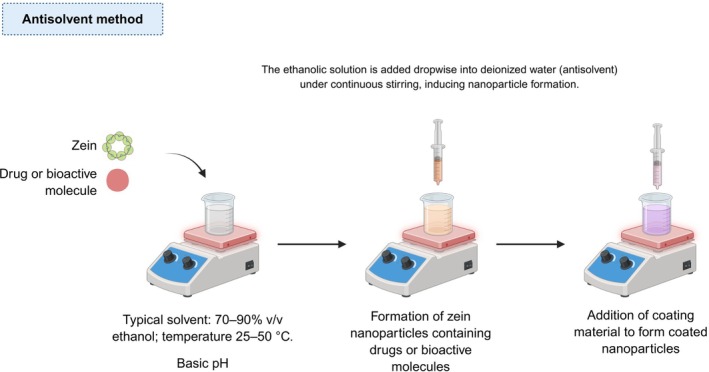
Illustration of the antisolvent synthesis method, the most commonly used approach for the production of ZNPs.

Sometimes cited as nanoprecipitation, this method involves the addition of a non‐solvent (antisolvent) to a solute dissolved in a solvent, leading to the formation of a supersaturated solution and subsequent precipitation. In this methodology, the characteristics of the nanoparticles depend on factors such as agitation speed, volume, and injection rate of the antisolvent into the solution [108]. As shown in Figure 4, its advantages stem from its simplicity, the accessibility of reagents, and the lack of expensive equipment, making it easily reproducible with a relatively short duration.

### 3.2 Desolvation

The desolvation method is a two‐step process involving solution purification and drying, resulting in a suspension of solid nanoparticles. Initially, zein is dissolved in ethanol by stirring at room temperature. Purified water is then added to the solution, leading to the formation of nanoparticles, followed by the evaporation of ethanol. The suspension is purified using tangential flow filtration to remove impurities. For nanoparticle coating, a solution of the coating material is added to the purified nanoparticle suspension, and the mixture is incubated under magnetic stirring at room temperature. Finally, the nanoparticle suspension undergoes drying in a spray‐dryer under controlled conditions, including inlet and outlet temperature, air pressure, pumping rate, aspirator, and airflow [10, 72, 109]. This method offers several benefits, such as precise control over nanoparticle size, high purity through effective filtration, and scalability for large‐scale production.

### 3.3 Electrostatic Deposition

In the electrostatic deposition technique, zein is dissolved in a suitable solvent and exposed to an electrostatic field, leading to the formation of nanoparticles. Coated zein nanoparticles (ZNP) encapsulating drugs can be prepared using this method at a controlled pH 4.0. The process begins by preparing a drug‐zein solution in ethanol, which is then added to water at pH 4.0 under continuous stirring. The organic solvent is subsequently evaporated, and additional water (pH 4.0) is introduced to achieve the desired final volume. The resulting mixture is filtered and gradually combined with a coating solution while stirring is maintained throughout the procedure [110]. This approach ensures the functionalization and physicochemical stability of the nanoformulation. Characterization studies confirmed the successful encapsulation of lutein within the zein polymer matrix, facilitated by electrostatic interactions, hydrophobic forces, and hydrogen bonding. This method yielded stable nanoparticles with well‐encapsulated lutein, optimizing drug delivery efficiency [77].

## 4 Materials Used as Coating of ZNPs

Biopolymer coatings play a crucial role in improving the stability and functionality of core‐shell zein nanoparticles. These coatings enhance pH stability, prevent aggregation, and ensure long‐term storage stability. Additionally, they provide protection against environmental stresses like heat, improving resistance to degradation. The coatings also increase encapsulation efficiency, enhancing the nanoparticles' drug delivery capabilities.

Overall, applying biopolymer coatings to zein nanoparticles not only enhances their stability and functionality but also broadens their potential applications, especially in drug delivery systems. A large number of materials are used for coating, as shown in Table 1.

### 4.1 Chitosan

Chitosan, a natural polysaccharide derived from chitin found in crustaceans like crabs and shrimp, exhibits remarkable properties such as biocompatibility, biodegradability, and the ability to form films and coatings. When used as a nanoparticle coating, it enhances stability, controls release, improves solubility and increases compatibility. Chitosan's coating also prevents the interaction or adsorption of encapsulated compounds with undesirable surfaces, ensuring efficient delivery to target sites. It is particularly valuable in oral nanocarriers due to its resistance to acidic stomach pH and mucoadhesive properties. This coating improves the storage stability and digestion of zein nanoparticles (ZNPs), playing a crucial role in promoting the sustained release of bioactive substances [103, 111].

Additionally, chitosan can expand intercellular junctions, facilitating transport across the intestinal epithelium and enhancing bioavailability. It also prolongs release in simulated gastrointestinal fluids and contributes to the mucoadhesive properties of ZNPs by promoting mucin adsorption on their surfaces [85].

### 4.2 Pectin

Pectin is one of the most studied polysaccharides for coating ZNPs and can be used to manufacture various nanoparticles such as core‐shell, microspheres, hydrogel spheres, and gels. Still, its wide use is also due to its biodegradability, safety, stabilization capacity, and low cost. This polysaccharide can be adsorbed on the surfaces of ZNPs and help to protect delivery systems and the material contained therein [76, 78].

The use of pectin is widely studied as a coating to promote more effective drug delivery systems and also hydrophilic polyphenols in nutraceutical formulations and food supplements. It can be absorbed on the surface of ZNPs and prevent their aggregation in aqueous solutions [38, 54].

### 4.3 Caseinates

Caseinate coating in ZNPs offers significant benefits, including improved particle stability, enhanced bioavailability, and controlled drug release. These properties make the formulation suitable for potential oral administration, while also serving as an electrostatic stabilizer that enhances the pharmacokinetic profile. By allowing for a lower dosage rate, caseinate improves drug tolerability and patient adherence to therapy. Additionally, caseinate's stabilizing and emulsifying effects contribute to the creation of antimicrobial films, with a water barrier and the capability for controlled release of thymol [57, 88]. These features highlight the value of caseinate as an effective coating material for drug delivery systems and biomedical applications.

### 4.4 Dextran

The nano complex of ZNPs carrying drugs and coated with dextran demonstrated improved drug stability, efficacy, and bioactivity. Dextran provided enhanced protection against degradation in the gastric environment, enabling controlled release of the active compound and enhancing its antioxidant activity [17]. Additionally, ZNPs coated with Dextran sulfate (ZDSNPs) exhibited a spherical structure formed through electrostatic attractions, hydrogen bonds, and hydrophobic interactions. Also, the presence of dextran sulfate reduced the surface hydrophobicity of the nanoparticles, altering the secondary structure of zein. Formulations with this type of coating exhibited excellent encapsulation efficiency, greater storage stability, and improved bioavailability, highlighting the benefits of using Dextran sulfate as a coating for enhancing the performance of bioactive‐loaded nanoparticles [20, 65].

### 4.5 Peg

PEGylation of ZNPs offers several key benefits, particularly in improving the performance and stability of drug delivery systems. Coating ZNPs with PEG enhances size control, encapsulation efficiency, and storage stability, while improving in vitro drug delivery, especially for hydrophobic drugs like hypericin used in photodynamic therapy. PEGylation also promotes passive targeting to tumor sites, increasing drug bioavailability, prolonging release, and enhancing antitumor efficacy. In addition, PEG coating improves physical and serum stability while ensuring hemocompatibility and better pharmacokinetics compared to other formulations [37, 101].

Furthermore, PEG‐coated ZNPs demonstrate superior blood glucose‐lowering effects and longer‐lasting hypoglycemic activity, with better mucus permeation and enhanced intestinal motility. For oral insulin delivery, PEG significantly boosts hypoglycemic efficacy and bioavailability, facilitating deeper diffusion through mucus layers and improving absorption at the intestinal surface. These advantages make PEGylation an essential modification for enhancing the therapeutic potential of ZNP [72, 73].

### 4.6 Saccharides

To reduce hydrophobic attraction and enhance the stability of zein nanoparticles, polysaccharides have been investigated to improve the surface of nanoparticles.

Soluble soybean polysaccharide (SSPS), a by‐product of tofu and soybean protein production, is water‐soluble, heat‐stable, and negatively charged. It can prevent pH‐dependent aggregation, whereas zein/SSPS composite nanoparticles remain stable over a broader pH range (5.0–7.0), indicating enhanced stability due to SSPS interaction. The addition of SSPS prevented heat‐induced aggregation of ZNPs and sustained stability during storage across pH conditions [46].

Alginic acid oligosaccharide (AOS) can be used to coat loaded ZNPs leveraging its high water solubility, stability, and diverse biological activities, making it suitable for drug delivery, functional food, agriculture, and animal development. Stability studies revealed that zein‐AOS complex nanoparticles are an excellent delivery system within the pH range of 4–9, showcasing remarkable thermal stability with minimal changes in size, PDI, and charge after exposure to temperatures between 30°C and 90°C for 30 min [18].

Pectin has been demonstrated to have the most efficiency in zein stabilization. Therefore, small‐sized polysaccharide (ESP) obtained from the precipitation process of pectin may be used as a stabilizer for zein self‐assembly during the preparation process. Zein‐ESP nanoparticles show a high efficiency, loading capacity, and bioaccessibility, suggesting that zein‐ESP can be applied as a delivery system for industrial food and drugs [24].

Tremella polysaccharide (TP), extracted from *Tremella fuciformis*, has been investigated for its anticancer, anti‐inflammatory, and immune regulation activities. Due to its non‐toxicity, TP is also demonstrated as a potential molecule to be applied in the food and pharmaceutical industry. The use of TP as a natural stabilizer can enhance the stabilization of zein nanoparticles, improving the encapsulation and delivery performance of various compounds. Furthermore, TP interacts with the surface of zein NPs, improving the hydrophilicity of zein and demonstrating a higher encapsulation efficiency, physical stability, and resistance [19].

### 4.7 Others

#### 4.7.1 Conjugates

ZNPs containing drugs were synthesized by conjugating mannosamine (GM) to the surface of the nanoparticles using a simple incubation process. This coating method enhanced the particles' residence time in the gut, improving oral immunization by allowing the drug to stay longer in the digestive tract [74]. Similarly, ZNPs were coated with Gantrez AN‐thiamine conjugates, which imparted mucus permeability properties to the nanoparticles, facilitating their passage through the protective mucus gel layer to reach the intestinal epithelium. The coating formed a continuous hydrophilic corona, improving the stability and consistency of the nanoparticles [69]. Additionally, the Gantrez AN‐thiamine coating increased the intestinal absorption of insulin, making it a promising method for oral insulin delivery [71]. ZNPs coated with the Gantrez‐mannosamine conjugate provided gastroprotection, preventing the premature release of the active ingredient in acidic conditions and enabling a controlled release in neutral or basic environments. This method improved the distribution and immunogenicity of Outer‐Membrane Vesicles, enhancing their effectiveness as oral vaccines [42, 93].

These coating strategies demonstrate significant advantages in enhancing bioavailability, stability, and targeted delivery for oral applications.

#### 4.7.2 Complexes

A core‐shell nanocomposite was developed using polydopamine (PDA) and casein to improve polyphenol delivery systems' stability and antioxidant activity. PDA's hydrophilicity, stability, and biocompatibility, along with its chemical structure similar to polyphenols, made it an ideal mediator for creating stable colloidal particles. Additionally, casein, a water‐soluble amphiphilic biopolymer, was used as an electrostatic and steric stabilizer, enhancing the colloidal stability of the particles [102].

A pH‐responsive delivery system was developed using tannic acid (TA)‐metal coordination networks on coated ZNPs. This system demonstrated the ability to release encapsulated drugs in a pH‐sensitive manner. The TA‐coated nanoparticles, coordinated with metals like FeIII, AlIII, or EuIII, showed excellent pH sensitivity and low cytotoxicity in vitro, making them suitable for targeted anticancer drug delivery [81, 82].

These methods highlight the advantages of using the combinations of two types of coating materials for enhancing nanoparticle stability, controlled release, and targeted delivery in therapeutic applications.

## 5 Biological Activities

After confirming the physicochemical stability of the nanoparticles, considering the synthesis method, coating material, particle size, and overall system viability, studies were selected to evaluate their biological activities. In vitro and in vivo models demonstrating antioxidant and antimicrobial effects, as well as selective activity against cancer cells, were prioritized to assess the advantages of coated nanoparticles, including improved selectivity, reduced adverse effects, prolonged release, low toxicity, and enhanced biological activity.

### 5.1 In Vitro

Exposure to nanoparticles can trigger various forms of toxicity, including the induction of reactive oxygen species, activation of autophagy, and other adverse cellular mechanisms. Moreover, before advancing to more complex experimental models, it is essential to obtain data that confirms the safety, selective toxicity (such as toward cancer cells), and overall feasibility of proceeding with the nanoparticles to subsequent stages of investigation [112].

To achieve this, a wide range of in vitro assays is available, involving different cell types and antioxidant activity tests. These assays can be used as a screening step to identify nanoparticles with greater potential and applicability.

#### 5.1.1 Antioxidant

Antioxidant studies of coated ZNPs are essential for exploring their potential as delivery systems for bioactive compounds with antioxidant properties. These studies are instrumental in assessing how nanoparticles preserve and enhance the antioxidant properties of encapsulated compounds, thereby broadening their applications in food, cosmetics, and pharmaceutical products [17, 26].

Studies evaluating the antioxidant properties of zein nanoparticles are widely conducted due to their practicality, simplicity, and ability to provide valuable insights into the protective potential of ZNPs toward antioxidant compounds. Such systems can enhance antioxidant responses by reducing reactive oxygen species (ROS), increasing the activity of antioxidant enzymes, improving nanoparticle stability, and promoting controlled release [113]. These studies are summarized in Table 2.

**TABLE 2:** Summary of studies involving ZNPs and antioxidant experiments.

Test conducted	Drug/coating	Results	Applicability of study	Author
DPPH	Curcumin/ethyl cellulose	− Nanoparticles maintained approximately 50% antioxidant activity after 6 months of formulation. − Free drug showed antioxidant activity lower than 20%.	Food formulations Drug delivery	Hasankhan et al. [59]
	Tanshinone/pectin	− Higher antioxidant in T/Z NPs: Tanshinone in lipophilic T/Z NPs showed stronger DPPH* scavenging (77.35%) than in T/Z/P NPs (72.04%). − Hydrophilic pectin coating in T/Z/P NPs slowed DPPH* penetration, limiting immediate antioxidant activity. − Z/P NPs without tanshinone still exhibited moderate antioxidant activity (~45%) due to zein's inherent properties.	Antioxidant	Elmizadeh et al. [56]
	Curcumin/Alginate and NaCas	− Nanoencapsulation significantly enhanced curcumin's antioxidant activity, as shown by SC50 values. − Ethanol‐dissolved curcumin had the worst antioxidant activity (SC50 = 8.79 μg/mL), equivalent to 67% of vitamin C (SC50 = 5.90 μg/mL). − Nanoencapsulated curcumin (SC50 = 2.65 μg/mL) was 3.32 times stronger than vitamin C. − SA/SC/Z nanoparticles effectively improved curcumin's antioxidant capacity by enhancing hydrophilicity and radical scavenging.	Drug delivery Antioxidant enhancement	Liu et al. [84]
	Thymol/sodium caseinate	− Nanoparticles without thymol showed < 20% DPPH* scavenging activity. − As the thymol‐to‐zein ratio increased from 0% to 40%, the DPPH* scavenging activity increased from 25% to 52%.	Antioxidant preservation in packaging Drug delivery	Kang‐Kang et al. [57]
	Curcumin/chitosan	− Cur/zein–HTCC nanoparticles showed enhanced antioxidant activity. − At a 1:1 zein–HTCC ratio, DPPH scavenging increased by: ○ 13.3% (pasteurization at low temperature) ○ 26.9% (pasteurization at high temperature) ○ 29.0% (UV exposure) − Encapsulation improved stability against heat and UV degradation.	Drug delivery Antioxidant therapy	Liang et al. [89]
	Curcumin/dextran	− Antioxidant activity increased with concentration for both free and encapsulated curcumin. − Encapsulated curcumin had higher DPPH radical scavenging efficiency than the free form. − Dextran‐zein nanoparticles enhanced curcumin's antioxidant capacity by protecting its structure. − Encapsulation reduced heat and light degradation, allowing lower curcumin doses for DPPH* reduction.	Drug delivery Antioxidant enhancement	Albogamy et al. [17]
	Naringin/Glutamine (Gln)‐modified chito‐oligosaccharides	− ZN NPs (without GCS): ~50% free radical scavenging at 500 μg/mL − GZN NPs (with GCS): > 50% scavenging at 100 μg/mL − Nar solution (free form): requires 25 mM (~6810 μg/mL) to reach 50% scavenging	Anti‐obesity therapy	Guo et al. [40]
	Quercetin/chitosan and shellac	− Quercetin‐loaded NPs showed greater DPPH* and ABTS** radical scavenging capacity than free quercetin, indicating improved antioxidant potential. − ZS and ZSC NPs exhibited notable antioxidant properties, likely due to the inherent antioxidant capacity of zein and chitosan. − Chitosan‐coated NPs had lower radical scavenging efficiency compared to uncoated NPs	Antioxidant	Liu et al. [51, 52]
ABTS+	Folic acid and caffeic acid/chitosa	− Antioxidant capacity of CS‐HZ before irradiation was 9.0 μg/mL VC − CS‐HZ/CA (56.9 μg/mL VC) showed weaker antioxidant capacity than the CA control (61.6 μg/mL VC), suggesting encapsulation partially masked the antioxidant capacity. − Ranking of antioxidant capacity of particles: CS‐HZ/CA > CS‐HZ/FA + CA > CS‐SZ/CA > CS‐SZ/FA + CA, according to the encapsulation efficiency (EE) of CA. − The more CA released, the stronger the antioxidant capacity. − Antioxidant capacity increased after 240 min due to the higher antioxidant capacity of CA's photoproduct, esculetin, compared to CA	Cancer treatment	El‐Lakany et al. [101]
	Quercetin/soluble soybean polysaccharide	− Zein, SSPS, and zein/SSPS nanoparticles neutralized 5.8%, 4.95%, and 9.13% of ABTS^+^, respectively. − Encapsulation of *Q* significantly (*p* < 0.05) enhanced its antioxidant activity compared to the free form	Antioxidant Cancer treatment	Li et al. [46]
DPPH, ABTS+, and FRAP	Curcumin/carboxylic curdlan	DPPH*: − Free curcumin had the lowest IC50 (7.23 μg/mL), outperforming encapsulated curcumin (106.3–174.3 μg/mL) and the positive control (33.0 μg/mL). − Among encapsulated samples, Cur‐24‐ZC showed the best antioxidant efficiency (IC50 = 106.3 μg/mL), linked to high EE and better dispersibility. ABTS^+^: − ABTS radical scavenging followed the DPPH trend, with significant differences between free and encapsulated curcumin (*p* < 0.05). FRAP: − Free curcumin had a higher FRAP value (~2005.8 μmol Fe^2+^/g) than encapsulated forms and the positive control. − Cur‐24‐ZC had the highest FRAP value (~200.6 μmol Fe^2+^/g) among encapsulated samples, supporting DPPH and ABTS results.	Antioxidant	Yu et al. [26]
DPPH, TEAC, and FRAP	Propolis/chitosan	DPPH (200 μg/mL): − Propolis‐loaded zein/CMCS nanoparticles: 79.69% − Propolis in ethanol: 88.01% propolis in water: 16.62% − Empty zein/CMCS nanoparticles: 44.04% TEAC*: − Propolis‐loaded zein/CMCS nanoparticles: 7902.12 ± 299.78 μmol Trolox/g − Propolis in ethanol: 8522 ± 692.62 μmol Trolox/g − Empty nanoparticles exhibited 2022 ± 152.23 μmol Trolox/g, suggesting zein/CMCS functional groups contribute to antioxidant activity. FRAP: − Encapsulated propolis: 2647.01 ± 174.57 μmol Fe^2+^/g − Propolis in ethanol: 611.34 ± 32.6 μmol Fe^2+^/g − Encapsulation enhanced antioxidant activity by protecting propolis and improving its interaction with Fe^3+^‐TPTZ.	Antioxidant applications in food and pharmaceuticals	Zhang et al. [45]
DPPH and ABTS+	Quercetin/gelatin and carboxymethyl starch	DPPH: − Free quercetin (in ethanol): 82.8% − Zein nanoparticles (blank): low but present due to sulfur‐containing amino acids and tryptophan − Zein‐Que NPs: increased compared to free quercetin − Zein/Gel/CMS‐Que NPs: highest activity, superior to Zein‐Que NPs, due to the synergistic effect of CMS, gelatin, and zein ABTS: − Free quercetin: 65.2% − Zein/Gel/CMS‐Que NPs: higher than Zein‐Que NPs, attributed to a favorable microenvironment for hydrogen and electron donation − Zein and Zein/Gel/CMS (blank): slight activity, attributed to amino acids and minerals present	Antioxidant therapy	Zhang et al. [49]
	Quercetin/auricularia cornea Ehrenb polysaccharides	DPPH: − ZAQ‐4 NPs showed higher radical scavenging than free quercetin (F‐Q) before GIT (IC50: 36.07 vs. 41.66 μg/mL) and after simulated GIT (48.8% vs. 29.07%). ABTS: − ZAQ‐4 NPs had greater scavenging than F‐Q before GIT (IC50: 114.5 vs. 132.4 μg/mL) and after GIT (37.62% vs. 16.69%).	Anti‐inflammatory	Wang et al. [100]
	Quercetin/Shellac	DPPH: − Encapsulated quercetin NPs > free quercetin in radical scavenging. − ZS and ZSC NPs showed inherent antioxidant activity. − Chitosan‐coated NPs had lower immediate scavenging due to slower quercetin release. ABTS: − Encapsulated quercetin NPs > free quercetin in radical scavenging. − ZS and ZSC NPs contributed to antioxidant activity. − Chitosan coating reduced the rate of ABTS radical scavenging.	Antioxidant	Liu et al. [51]
	GuavinosideB/caseinate	DPPH: − GUB‐Z‐N NPs showed strong radical scavenging, slightly lower than free GUB; blank Z‐N NPs had negligible activity. ABTS: − GUB‐Z‐N NPs exhibited higher scavenging than free GUB, especially at higher concentrations; blank Z‐N NPs also showed some activity due to NaCas.	Antioxidant	Yang et al. [35]
	Curcumin/quaternary ammonium chitosan	DPPH: − CUR‐ZE‐SC and CUR‐ZE‐SC@HACC NPs showed higher scavenging (57.2% and 65.9%) than free CUR (48.8%); blank carriers also contributed slightly (~8%–15%). ABTS: − CUR‐ZE‐SC@HACC NPs exhibited strongest scavenging (76.4%), surpassing CUR‐ZE‐SC (54.3%) and free CUR (39.8%); blank carriers showed moderate activity (~9%–35%).		Liu et al. [52]
	Resveratrol or tannic acid/pectin	DPPH: − Tannic acid‐loaded nanoparticles had higher antioxidant activity (SC50 = 3.02 μg/mL) than ascorbic acid (SC50 = 5.17 μg/mL). − Resveratrol‐loaded nanoparticles (SC50 = 9.70 μg/mL) outperformed free resveratrol (SC50 = 35.81 μg/mL). − Co‐loaded TA + resveratrol nanoparticles (SC50 = 4.41 μg/mL) showed a synergistic effect, enhancing resveratrol's activity. ABTS^+^: − No significant difference was found between encapsulated TA and co‐encapsulated TA + resveratrol.	Cancer treatment Antioxidant enhancement	Liang et al. [55]
	Guavinoside B/sodium caseinate	DPPH: − GUB‐Z‐N NPs demonstrated antioxidant activity comparable to ascorbic acid, with an IC50 of 20.89 μM. ABTS^+^: − Encapsulated GUB showed significantly higher ABTS + scavenging activity than free GUB	Antioxidant	Yang et al. [35]
	Resveratrol/chitosan	ABTS^+^: − Resveratrol neutralized ABTS^+^· more effectively than HZ and HZ‐CH particles. DPPH: − Encapsulation in HZ and HZ‐CH improved DPPH· radical neutralization by increasing solubility.	Antioxidant therapy Drug delivery	Khan et al. [103]

* DPPH: 2,2‐diphenyl‐1‐picryl‐hydrazyl/ABTS+: 2,2′‐Azinobis‐3‐Ethylbenzthiazolin‐6‐Sulfonic Acid/FRAP: ferric reducing antioxidant ability/TEAC: Trolox equivalent antioxidant capacity.

#### 5.1.2 Antimicrobial

The development of new antimicrobial drugs is essential due to the high incidence of infections, particularly in hospital settings, and the increasing resistance to existing treatments. Research into novel therapeutic options is crucial for effectively combating resistant microorganisms. Additionally, many antimicrobial agents cause significant adverse effects, leading to poor patient adherence to treatment [114].

As shown in Table 3, nanotechnology presents innovative solutions by enabling the combination of drugs with different mechanisms of action, enhancing their effectiveness against pathogens. This approach also facilitates the development of potent alternatives for infections requiring multiple doses or complex treatment regimens, ultimately improving patient compliance [115].

**TABLE 3:** Summary of studies involving ZNPs and antimicrobial experiments.

Microorganism	Drug/coating	Results	Applicability of study	Author
*E. coli* and *Salmonella*	Thymol/sodium caseinate	− Films without thymol (ZP0) and with low thymol content (ZP1) showed no significant inhibition halos against tested pathogens, with bacterial colonies growing on ZP0 films. − Films with zein‐SC nanoparticles and thymol‐to‐zein ratios of 30%–40% (ZP3 and ZP4) exhibited visible inhibition halos, indicating antimicrobial activity. − The diameter of inhibition halos increased with higher thymol loading, ranging from 16 to 18 mm, demonstrating enhanced antimicrobial efficacy.	Development of antimicrobial packaging films	Kang‐Kang et al. [57]
*S. aureus* , *E. coli* and *Candida albicans*	Curcumin/Dextran	− DSZCNPs demonstrated significant antibacterial activity, with CFU reduction ranging from 53.74% to 97.53% against gram‐positive bacteria and 52.35%–71.42% against gram‐negative bacteria, compared to free curcumin. − Both free curcumin and DSZCNPs showed similar effects against *Candida albicans* (~50% CFU reduction). − Thymol (free and encapsulated) showed significant inhibition only against *S. aureus* , with no effect on other bacteria or the fungus. − Thymol encapsulated in CHC‐SC coated nanoparticles was effective against *S. aureus* , showing significant inhibitory activity after 16 h. − The SC/CHC mass ratio of 1:4 in encapsulated thymol nanoparticles exhibited the longest and most significant inhibitory effect throughout the testing period (*p* < 0.05).	Drug delivery systems, enhanced antimicrobial activity	Albogamy et al. [17]
*S. aureus* , *E. coli* , *Pseudomonas aeruginosa* and *Candida albicans*	Thymol/sodium caseinate and chitosan	− DSZCNPs demonstrated significant antibacterial activity, with CFU reduction ranging from 53.74% to 97.53% against gram‐positive bacteria and 52.35%–71.42% against gram‐negative bacteria, compared to free curcumin. − Both free curcumin and DSZCNPs showed similar effects against *Candida albicans* (~50% CFU reduction). − Thymol (free and encapsulated) showed significant inhibition only against *S. aureus* , with no effect on other bacteria or the fungus. − Thymol encapsulated in CHC‐SC coated nanoparticles was effective against *S. aureus* , showing significant inhibitory activity after 16 h. − The SC/CHC mass ratio of 1:4 in encapsulated thymol nanoparticles exhibited the longest and most significant inhibitory effect throughout the testing period (*p* < 0.05).	Antimicrobial applications in wound care and food preservation	Zhang et al. [86]
*S. aureus* and *E. coli*	Tannic acid/Copper‐doped bioactive glasses and Sodium carboxymethyl cellulose	*S. aureus* : − Zein/CMC + CuBG: No inhibition observed. − Zein/CMC + CuBG + TA: Significant reduction in metabolic activity. − TA release: 51 ± 11 μg/mL. *E. coli* : − Zein/CMC + CuBG: No inhibition observed. − Zein/CMC + CuBG + TA: Significant reduction in metabolic activity. − TA release: 51 ± 11 μg/mL.	Antibacterial therapy	Hadzhieva et al. [63]
*Pseudomonas aeruginosa* and *Klebsiella pneumoniae*	Ceftazidime and tobramycin/chitosan	Antibacterial activity (MIC/MBC): − CAZ: MIC 12.5–> 50 μg/mL; MBC 25–> 50 μg/mL. − TOB: MIC 6.25–12.5 μg/mL; MBC 12.5–50 μg/mL. − CAZ–ZNP–CH: MIC 3.12–12.5 μg/mL; MBC 12.5–25 μg/mL. − TOB–ZNP–CH: MIC 1.56–3.12 μg/mL; MBC 6.25–25 μg/mL. − CAZ–TOB–ZNP–CH: MIC 0.19–3.12 μg/mL (CAZ) and 0.15–2.40 μg/mL (TOB); MBC 1.56–6.25 μg/mL (CAZ) and 1.21–4.87 μg/mL (TOB). − ZNP–CH showed no antibacterial activity (> 50 μg/mL). Antibiofilm activity (inhibition + eradication): − Biofilm inhibition (MIC → MIC/16): CAZ (5%–80%), TOB (4%–88%), CAZ–ZNP–CH (49%–93%), TOB–ZNP–CH (53%–100%), CAZ–TOB–ZNP–CH (69%–100%). − MBIC: Encapsulated drugs < free drugs; CAZ–TOB–ZNP–CH shows 10–35× lower MBIC values than single‐drug NPs. − Biofilm eradication (MIC → 16 × MIC): CAZ (18%–68%), TOB (27%–79%), CAZ–ZNP–CH (40%–81%), TOB–ZNP–CH (43%–84%), CAZ–TOB–ZNP–CH (58%–92%). − MBEC: Encapsulated drugs < free drugs; CAZ–TOB–ZNP–CH shows 8–300× lower MBEC values. − ZNP–CH showed no antibiofilm activity.	Antibacterial therapy	Campos et al. [96]

Moreover, antimicrobial nanoparticles have applications beyond healthcare, particularly in the food industry, where they can extend the shelf life of products by preventing microbial contamination. Their incorporation into packaging materials and food preservation systems helps maintain quality for longer periods, reducing waste and ensuring safer consumption [116].

#### 5.1.3 Cell Culture

In vitro cell‐based experiments are essential for developing and evaluating new drug therapies. These studies enable researchers to assess the effectiveness and selectivity of nanosystems, such as ZNPs, for cancer treatment while minimizing adverse effects. They also help determine the optimal concentration of stabilizing surfactants to ensure the safety and stability of these systems in experimental settings [101].

Moreover, in vitro models provide valuable insights into the dual behavior of certain drugs. At lower concentrations, these compounds may act as antioxidants, protecting cells from damage, while at higher doses, they can exhibit significant anticancer properties [102]. Lipophilic compounds, in particular, tend to accumulate more easily within cells, leading to increased cytotoxic effects. Such studies are crucial for refining drug formulations, evaluating their safety, and optimizing therapeutic potential before progressing to clinical trials [59].

Table 4 summarizes the main findings from studies on coated ZNP. Overall, these studies have demonstrated significant anticancer properties in cell lines. Additionally, their safety, biocompatibility, immunogenicity, and antioxidant properties have been explored to a lesser extent.

**TABLE 4:** Summary of studies Involving ZNPs and cell culture experiments.

Cell type used	Drug/coating	Results	Applicability of study	Author
Ehrlich ascites carcinoma, MCF‐7*, 4T1*	Exemestane and luteolin/PEGylated phospholipids and lactoferrin	− PEGylated nanospheres improve cytotoxicity against MCF‐7 (IC50 = 6.6 μg/mL) and 4TI breast cancer cells (IC50 = 5.07 μg/mL). PEGylation improves antitumor efficacy and pharmacokinetics compared to free‐drug	Hepatocellular carcinoma treatment	El‐Lakamy et al. [101]
HepG2*	Hypericin/PEG or NaCas	− In dark conditions, both free and zein‐loaded hypericin showed no significant toxicity to HepG2 and L929 cells. However, upon irradiation, cell viability decreased in a dose‐dependent manner, with the strongest cytotoxic effects observed at 1.5 J/cm^2^. − The PEGylated zein formulation (Z‐PEG) demonstrated the highest cytotoxicity in HepG2 cells at 1.5 J/cm^2^ while maintaining a safer profile in non‐cancerous L929 cells, indicating improved cancer cell targeting and reduced off‐target effects.	Hepatocellular carcinoma treatment	Abdelsalam et al. [37]
	Curcumin/Dextran	− Great stability in time (100 days) and protects from degradation in gastric environment with controlled curcumin release − Curcumin concentrations: 10–80 μg/mL − DSZCNPs showed higher cytotoxicity than free curcumin at low concentrations (10, 20, 40 μg/mL). IC50 values: − Free curcumin: 50 μg/mL. DSZCNPs: 13 μg/mL → more effective.	Hepatocellular carcinoma treatment	Albogamy et al. [17]
	Doxorubicin/Chitosan and metal–tannic acid	− DOX‐loaded metal–TA‐Coated NPs had lower cytotoxicity than DOX‐loaded Zein/CMCS NPs IC50 values: − DOX‐NPs: 2.36 ± 0.18 μg/mL − DOX‐NPs‐TA/Cu^2+^: 2.91 ± 0.12 μg/mL − DOX‐NPs‐TA/Ca^2+^: 2.67 ± 0.28 μg/mL − DOX‐loaded zein/CMCS NPs exhibited higher cytotoxicity than metal–TA‐coated NPs due to their faster drug release and efficient cellular uptake. − DOX‐loaded metal–TA‐coated NPs displayed a more controlled drug release profile, leading to a delayed but sustained cytotoxic effect.	Hepatocellular carcinoma treatment	Liang et al. [68]
	Tannic acid and resveratrol/pectin	− Encapsulated tannic acid was the most cytotoxic presenting an IC50 = 8.36 ± 0.22 μg/mL, making it 6× more toxic than encapsulated resveratrol. − Co‐encapsulation of tannic acid and resveratrol resulted in intermediate toxicity, presenting an IC50 = 10.07 ± 0.23 μg/mL, similar to encapsulated tannic acid, suggesting that tannic acid played a dominant role in cytotoxicity.	Hepatocellular carcinoma treatment	Liang et al. [55]
	Gambogenic acid/polydopamine	− GNA@Zein‐PDA particles had the strongest cytotoxic effect. IC50 values: − Free GNA: 9.89 μg/mL − GNA@Zein NPs: 4.42 μg/mL − GNA@Zein‐PDA NPs: 1.59 μg/mL	Hepatocellular carcinoma treatment	Zha et al. [34]
A549*, K562*	Sodium deoxycholate (coating only)	− Adding surfactant sodium deoxycholate (1.25% w/v) provides high storage and thermal (up to 50°C) stability, resistance to pH alterations and freeze‐drying process. A549 cells: − Surfactant‐free zein nanoparticles induced cytotoxicity only after 72 h at 100 μg/mL and reduced cell viability by 25%–30% − Surfactant‐coated zein nanoparticles showed significant toxicity at 50 μg/mL after just 24 h. K562 cells: − Cell viability gradually decreases as the concentration of zein nanoparticles increases, with higher concentrations (> 10 μg/mL) exhibiting a more pronounced effect. − Nanoparticles incorporating surfactants (T80, PLX188, and SD) demonstrate greater toxicity compared to surfactant‐free formulations.	Safety evaluation of ZNPs in cancer treatments	Gagliardi et al. [91]
MCF‐7*, K562	Paclitaxel/sodium deoxycholate	− PTX‐loaded ZNPs enhanced cytotoxicity against cancer cells − Higher cytotoxicity observed in nanoencapsulated PTX compared to free PTX. − MCF‐7 cells exhibited a faster response to PTX‐loaded zein NPs, whereas K562 cells showed stronger inhibition only after 72 h. − At a concentration of 1 μM, cell viability reached its minimum point.	Breast cancer and leukemia treatment	Gagliardi et al. [94]
Caco‐2 cells*	Insulin/chitosan	After 24 h incubation: − IN‐Z‐CSA1.0%: 5.64% apoptosis. − IN‐Z‐CSA/CS0.2%: 7.36% apoptosis.	Cellular uptake studies for drug delivery	Ji et al. [61]
	Quercetin/Trimethylated chitosan	− Zein‐Q and TMC‐Zein‐Q enhanced Caco‐2 cell viability compared to free quercetin, indicating improved biocompatibility and reduced cytotoxicity. − TMC‐Zein‐Q significantly increased cellular uptake of quercetin, attributed to enhanced endocytosis induced by the TMC shell.		Dai et al. [48]
HeLa cells	Doxorubicin/Tannic acid	IC50 values: − Free DOX: 0.918 ± 0.05 μg/mL − DOX‐zein NPs: 0.939 ± 0.04 μg/mL − DOX‐zein/TA NPs: 1.087 ± 0.02 μg/mL − TA‐coated NPs showed slower DOX release, leading to reduced intracellular accumulation.	Cervical cancer treatment	Liang et al. [98]
RAW 264.7 macrophages*	Outer membrane vesicles obtained from Enterotoxigenic *Escherichia coli* serotypes/Gantrez‐mannosamine polymer conjugate	− The tested outer membrane vesicles (OMV) at concentrations ranging from 7.5 to 120 μg/mL did not exhibit cytotoxicity, as cell viability remained close to 100%. − OMVs at 10, 40, and 160 μg/mL significantly increased nitric oxide production in macrophages suggesting antigenic response	Immune response and macrophage interaction studies	Matías et al. [93]
A549, NIH‐3 T3 cells	Ellipticine/NaCas or polyethylene imine	A549: − Zein/SC‐EPT: 54%–81% cytotoxicity (0.49–7.81 μg/mL). − Zein/PEI‐EPT: 61%–92% cytotoxicity (0.49–7.81 μg/mL). − Control EPT (water, 7.81 μg/mL): ~60% cytotoxicity. NIH‐3T3: − EPT‐loaded nanoparticles increased toxicity to cancer cells but spared NIH‐3T3 cells.	Lung cancer treatment	Pourhossein et al. [62]
293*, A549 cells	Resveratrol/polydopamina and casein	− Zein‐polydopamine‐casein core–shell nanocomposites (ZPC) increase their uptake in cells compared to unmodified nanoparticles (from 2.79% to 97.66%). − The cytotoxicity of ZPCs was tested on 293 and A549 cells, and no toxicity was observed at any of the tested concentrations (10–1000 μg/mL). − The cytotoxicity of RES was assessed on A549 cells, with no inhibitory effect observed at concentrations below 100 μM. At higher concentrations (> 100 μM) − The cytotoxicity of RES‐ZPCs was evaluated on A549 cells. At higher RES concentrations (> 100 μM), cell viability decreased to approximately 60% at 200 μM. The cytotoxicity of RES‐ZPCs was lower than that of RES alone. ROS scavenging efficiency of RES‐ZPCs was 100% at 50 μM.	Biocompatibility and antioxidant studies	Shaomin et al. [102]
HT29 cells*	Curcumin/ethyl cellulose	− Cytotoxicity was evaluated at concentrations between 1.25 and 20 μg/mL^−1^ − The nanoparticle formulation (NPs of CU) exhibited lower cytotoxicity compared to free CU.	Colon cancer treatment	Hasankhan et al. [59]
NCM 460*, RAW 264.7	Magnolol/chondroitin sulfate	− Safe doses in both cell lines are 5–20 μM and 1–10 μM in NCM 460 and RAW 264.7 cells, respectively. − Significantly reduced the levels of TNF‐a, IL‐6, and IL‐1β (proinflammatory factors) and increased the level of IL‐10 (anti‐inflammatory factor) in NCM 460 and RAW 264.7 cells along with an increase in cellular uptake.	Ulcerative colitis treatment	Wang et al. [39]
MCF‐7, SKOV‐3 cells	Docetaxel/soy lecithin and chitosan	MCF‐7: − Unloaded FZLC: Showed no cytotoxic effects at any concentration up to 48 h. − Free DTX: Induced moderate cytotoxicity, with only a slight increase as the concentration increased. − DTX/FZLC NPs: Exhibited significantly higher cytotoxicity than raw DTX, particularly at higher concentrations. SKOV‐3: − Unloaded FZLC: Did not affect cell viability at any tested concentration. − Free DTX: Displayed limited cytotoxic effects, with only a slight increase at higher concentrations. − DTX/FZLC NPs: Showed a pronounced increase in cytotoxicity compared to raw DTX, especially at elevated doses and prolonged incubation.	Cancer treatment (breast cancer, ovarian cancer)	Wu et al. [66]
NCM460 cells*	Curcumin/Dextran sulfate	− The IC50 values for ZNPs and ZDSNPs were 9.1 × 10^4^ μg/mL and 2.1 × 10^5^ μg/mL, respectively, indicating minimal toxicity.	Biocompatibility study	Yuan et al. [20]
	Lutein/sophorolipid	− Nanoparticles were tested at 100, 50, 25, 12.5, and 6.25 μg/mL, with lutein ranging from 0.82 to 0.05 μg/mL. − All formulations maintained viability above 80%, confirming low toxicity. − Free lutein showed slightly lower cell viability compared to nanoparticle formulations, especially at 100 and 50 μg/mL.	Biocompatibility and drug delivery studies	Yuan et al. [77]
C6 glioma cells*	Curcumin/dodecamer peptide (G23)‐functionalized polydopamine	− CUR‐ZpD NPs showed stronger inhibition of C6 glioma cells compared to free curcumin, likely due to enhanced cellular uptake. − At 5 μg/mL of curcumin, cell viability dropped to 32% with CUR‐ZpD NPs and 25% with CUR‐ZpD‐G23 NPs − CUR‐ZpD and CUR‐ZpD‐G23 NPs further suppressed colony formation, reducing survival to 18% ± 5% (CUR‐ZpD NPs) and 17% ± 6% (CUR‐ZpD‐G23 NPs) Cell migration was significantly reduced in curcumin‐treated cells compared to untreated controls: − Control: 75.0% ± 9.4% migration − Curcumin‐treated: 35.6% ± 8.7% migration − CUR‐ZpD NPs: 21.8% ± 6.6% migration − CUR‐ZpD‐G23 NPs: 23.9% ± 6.9% migration	Glioma treatment	Zhang et al. [21, 22, 23]
Caco‐2*, HT29‐MTX cells*	Insulin/PEG	− No cytotoxic effect was observed for either bare or PEG‐coated insulin‐loaded nanoparticles. − All tested concentrations (1.7–70 μg/mL of insulin) maintained cell viability after 24 h of incubation.	Intestinal drug delivery systems	Reboredo et al. [72]
L929, A549, HeLa, B16*	Paclitaxel/B16 cancer cell membrane	− Different concentrations of PTX concentrations: 4.50, 2.00, 0.89, 0.40, and 0.18 μg/mL Cell viability at minimum drug concentration: − A549: 87.11% − Hela: 71.4% − B16: 55.72% Cell viability at highest drug concentration: − A549: 50.22% − Hela: 54.04% − B16: 33.47% − L929 cell viability remained above 90%, indicating minimal cytotoxic effect and that CCM‐α‐Zein exhibits good biocompatibility with non‐tumor cells	Cancer treatment	Huang et al. [67]
HepG2 and AML12*	Celastrol/Hyaluronic acid	HepG2 IC50 values: − Free Cel: 14.34 μM − Cel/Zein NPs: 10.19 μM − Cel/Zein@HA NPs: 6.36 μM AML12 cells − Viability remained above 80% after 48‐h co‐incubation with Cel/Zein@HA NPs	Hepatocellular carcinoma treatment	He et al. [117]
HK‐2*	Resveratrol/Hyaluronic acid	− Cell viability of HK‐2 renal tubular endothelial cells treated with Zein/Res NPs and HA‐Zein/Res NPs was greater than 90% at a concentration of 200 μg/mL	Reduction of cisplatin‐associated nephrotoxicity	Ning et al. [118]
Caco‐2	Curcumin/Carboxymethylated corn fiber gum	− No cytotoxicity was detected at 20 mg/mL for CFG and all CMCFG samples, with cell viability remaining at ~100%. − Increasing concentrations (30–50 mg/mL) reduced viability in all groups, with CFG showing the greatest decrease while CMCFGs maintained higher viability. − Higher toxicity of CFG at 40–50 mg/mL was likely due to increased osmotic pressure compared to CMCFG solutions.	Hydrophobic nutrient delivery	Ma et al. [107]
MDA‐MB‐231*	Cinnamaldehyde and naringin/Lactoferrin and sodium caseinate	− Cell viability decreased in a concentration‐dependent manner for all treatments, with the lowest viability (~6%) observed when 40 μM DOX was combined with 25 μM dual drug‐loaded NPs. − Dual drug‐loaded NPs enhanced DOX cytotoxicity, showing lower IC50 values than blank NPs and the free‐drug combination. − Combination index analysis indicated that the DOX/dual drug‐loaded NP treatment achieved the strongest synergistic effect (CI = 0.968).	Enhance the gastric protection, bioavailability, and antioxidant efficacy	Mohamed et al. [15]
MCF‐7	Quercetin and Doxorubicin/Chitosan	− DOX showed strongly pH‐dependent release, with much higher release at pH 7.4 (~96%) than 6.8 (~68%), while QUE displayed similarly low release in both (~35%–38%). − In combined release, QUE exhibited a slight reduction, and pH 6.8 limited the diffusion of both drugs due to reduced hydrogel swelling. − OSAGX enhanced DOX cytotoxicity by 4.66×, OSAGC by 20.7×, while QNP alone showed no cytotoxic effect.	Localized breast cancer therapy	Da Paz Do Nascimento et al. [105]
HepG2	Quercetin/Alginate‐pectin	− Digested quercetin‐loaded nanoparticles significantly improved intracellular antioxidant defenses in H_2_O_2_‐treated HepG2 cells, with SOD activities markedly higher than those of digested quercetin in physical mixtures (PM), especially for ZQ5 and ZQ9. − CAT activity was also enhanced by the nanoparticles, reaching levels close to untreated control cells, while digested PM showed a smaller increase in enzyme activity. − These results indicate that nanoparticle delivery of quercetin effectively reduces oxidative stress and restores intracellular enzymatic antioxidant activities in HepG2 cells compared with quercetin in physical mixtures.	Nutraceutical bioavailability enhancement	Wan et al. [50]
RAW264.7	Astaxanthin/Chitosan‐α‐lipoic acid copolymer and sodium alginate	− HZ‐CL‐SA nanoparticles showed no significant cytotoxicity in RAW264.7 cells, indicating safe delivery of AST to target cells, while free AST was non‐toxic at 0.5–20 μg/mL but reduced viability at higher doses (> 20 μg/mL). − AST at 10 μg/mL significantly protected RAW264.7 cells from LPS‐induced inflammation, restoring cell viability to 97.7% compared with the LPS‐treated group. − AHZ‐CL‐SA nanoparticles effectively inhibited LPS‐induced inflammatory markers (NO, TNF‐α, IL‐1β, IL‐6) more strongly than free AST, with GSH‐pretreated nanoparticles showing enhanced anti‐inflammatory activity due to accelerated AST release.	Colon‐targeted nutraceutical delivery	Wang et al. [104]
PC3, DU154, and LNCa	Terbinafine/Dextran Sulphate	− TRB‐DSZN nanoparticles significantly enhanced antiproliferative activity in PC3 prostate cancer cells, reducing IC50 to 13.28 μg/mL compared with 163.4 μg/mL for free TRB and 45.66 μg/mL for blank DSZN NSs, while showing weak cytotoxicity in non‐cancerous EA.hy926 cells. − TRB‐DSZN NSs promoted cell cycle arrest in G0/G1 and S phases and increased apoptosis, as indicated by annexin‐V staining, compared with free TRB and blank DSZN NSs. − Treatment with TRB‐DSZN NSs upregulated pro‐apoptotic genes (CASP3, P53) and ROS production, while downregulating anti‐apoptotic genes (CDK1, CDK7, CDK9), demonstrating enhanced apoptosis and oxidative stress in PC3 cells compared with controls.	Prostate cancer therapy	Al‐Sawahli et al. [106]
MC3T3‐E1 (99072810‐1VL)	Tannic acid/Copper‐doped bioactive glasses and Sodium carboxymethyl cellulose	− Zein/CMC, zein/CMC/CuBG, and zein‐TA/CMC/CuBG coatings showed good cytocompatibility with MC3T3‐E1 preosteoblasts, as WST‐8 assays indicated no significant differences in cell viability after 24 h. − The addition of TA to CuBG‐containing coatings synergistically improved cell viability, with zein‐TA/CMC/CuBG performing better than zein/CMC/CuBG. − Fluorescence microscopy and SEM images confirmed that all coatings were non‐toxic after 72 h, with cells exhibiting well‐spread morphology and normal growth.	Bone regeneration	Hadzhieva et al. [63]
HepG‐2	Naringin/Glutamine (Gln)‐modified chito‐oligosaccharides	− GZN nanoparticles showed no cytotoxicity in HepG2 cells after 12 and 24 h, demonstrating good biocompatibility. − Treatment with GZN NPs alleviated insulin resistance in HepG2 cells, restoring glucose uptake to 72% of normal levels at 50 μg/mL. − GZN NPs reduced intracellular lipid accumulation in a concentration‐dependent manner, indicating potential to mitigate steatosis and abnormal lipid metabolism. − The nanoparticles also decreased ALT and AST levels and lowered intracellular triglycerides and total cholesterol, suggesting protective effects on liver metabolism and cellular fat regulation.	Food additives Pharmaceutical drug delivery	Guo et al. [40]
HT‐29	Colchicine/Eudragit S100	− Col‐Z nanoparticles exhibited concentration‐dependent cytotoxicity in HT‐29 cells, comparable to free colchicine, while colchicine‐free Z NPs showed no significant inhibition of cell viability. − Both Col‐Z NPs and free colchicine displayed lower cytotoxicity in HUVEC cells compared with HT‐29 cells, indicating selectivity toward cancer cells. − Fluorescence microscopy demonstrated time‐dependent cellular uptake of Z NPs, with higher intensity after 3 h compared to 1 h. − Col‐Z NPs showed low hemolytic activity (< 5%) and induced apoptosis in HT‐29 cells (42.9%), similar to free colchicine, confirming their hemocompatibility and anticancer potential.	Colorectal cancer treatment	Taymouri et al. [16]
WI‐38 fibroblasts, HSF	Fusidic acid/Lactoferrin	− LF‐ZF nanoparticles significantly enhanced the viability of WI‐38 fibroblasts, demonstrating the cytocompatibility of zein and the proliferative effects of lactoferrin (LF). − LF‐ZF‐NPs improved fibroblast adhesion compared to ZF‐NPs, likely due to LF's immunomodulatory effects and zein's interaction with tissue transglutaminase. − Scratch wound assays showed accelerated migration and proliferation of fibroblasts with LF‐ZF‐NPs, resulting in higher wound closure rates at 24 and 48 h. − These results indicate that LF‐ZF‐NPs may effectively promote fibroblast‐mediated wound healing, consistent with previous studies on lactoferrin in epithelial tissue repair.	Advanced chronic wound treatment.	Marey et al. [64]

* Ehrlich ascites carcinoma–Ehrlich ascitic carcinoma cells (tumor in mice)/MCF‐7—Human breast adenocarcinoma cells/4T1—Murine mammary carcinoma cells (mouse)/HepG2—Human hepatocellular carcinoma cells/A549—Human lung carcinoma cells/K562—Human chronic myelogenous leukemia cells/Caco‐2—Human colon adenocarcinoma cells/HeLa—Human cervical adenocarcinoma cells/RAW 264.7—Mouse leukemia‐derived macrophage cells/293—Human embryonic kidney cells (HEK293)/HT29—Human colon adenocarcinoma cells/NCM 460—Human colon epithelial cells/SKOV‐3—Human ovarian carcinoma cells/C6 glioma cells—Rat glioma cells/L929—Mouse fibroblast cells/NIH‐3T3—fibroblast/AML12—Normal liver cell/HK‐2—Human renal tubular epithelial cell/MDA‐MB‐231‐human breast adenocarcinoma/PC‐3 ‐ human prostate adenocarcinoma derived from bone metastasis/DU145 ‐ human prostate carcinoma derived from brain metastasis/LNCaP ‐ androgen‐sensitive human prostate adenocarcinoma derived from lymph node metastasis/MC3T3‐E1 ‐ mouse pre‐osteoblast cell line/WI‐38 ‐ normal human lung fibroblasts/HSF ‐ human skin fibroblasts.

### 5.2 In Vivo Models

In vivo studies validate and complement in vitro research by offering a comprehensive view of drug behavior in an organism, enabling the assessment of distribution, metabolism, and bioavailability across multiple organs [119]. Various models, including mammals, worms, and fish, are used to simulate how nanoparticles and drugs interact within living systems, offering valuable data on efficacy and potential adverse effects. These studies are critical steps in the research pipeline, bridging the gap between preclinical findings and clinical applications and ultimately ensuring the safety and effectiveness of new therapeutic strategies [120]. For this review, articles with concise and concrete data from in vivo studies were included, clearly elucidating the importance of the model in the work and future perspectives for clinical studies. In Table 5 we displayed the main outcomes of in vivo effects of ZNP in before mentioned species.

**TABLE 5:** Summary of studies involving ZNPs and in vivo experiments.

Model	Type	Drug/coating	Results	Applicability of study	Author
Mammalian	Wistar rats	Insulin	− Nanoencapsulated insulin began reducing blood glucose after 4 h, with a peak effect at 9 h and returning to baseline by 20 h. − Subcutaneous insulin had a faster onset, reaching its lowest blood glucose level within 2 h.	Oral drug delivery and diabetes treatment	Inchaurraga et al. [70]
		Insulin/PEG	− While subcutaneous insulin led to a rapid decrease in blood glucose (22% of initial levels within 2 h), I‐NP‐PEG provided a slower but sustained reduction, reaching a maximum decrease of 32% at 6 h, matching the glycemic levels of subcutaneous insulin. This suggests improved prolonged glucose regulation. − PEG‐coated insulin nanoparticles exhibited a 3‐fold increase in pharmacological availability (15% vs. 4.7% for bare nanoparticles) and a 2.5‐fold increase in oral bioavailability (10.2% vs. 4.2%). Additionally, I‐NP‐PEG resulted in higher and more prolonged plasma insulin levels compared to non‐PEGylated formulations, demonstrating improved absorption and efficacy.	Oral insulin delivery and bioavailability enhancement	Reboredo et al. [72]
		Insulin/Chitosan	− Subcutaneous insulin rapidly reduced blood glucose to 29.4% at 2 h, but levels returned to baseline by 8 h. − IN‐Z‐CSA/CS0.2% nanocomposites showed a more sustained reduction (38.8%) at 3 h, while IN‐Z‐CSA1.0% reached 49.4%. − Nanocomposites provided gradual plasma insulin release and sustained hypoglycemic effects for up to 8 h, unlike subcutaneous insulin. − No significant body weight changes or organ toxicity were observed, indicating safety for oral insulin delivery.	Enhanced insulin delivery in diabetic treatment	Ji et al. [61]
		Insulin/PEG	− Encapsulated insulin (IN‐Z‐CSA/CS0.2%) exhibited a slower, sustained hypoglycemic effect, peaking at 3 h and lasting up to 8 h. − Higher relative bioavailability (14.12%) compared to free insulin. − Gradual increase in plasma insulin levels, peaking at 3 h and remaining elevated longer than subcutaneous insulin. − Non‐toxic, with no significant changes in body weight or organ health in treated mice.	Potential treatment for diabetes and hypoglycemia	Reboredo et al. [73]
		None/Gantrez AN‐thiamine conjugate	− At 2 h post‐administration, nanoparticles were mainly found in the stomach and small intestine. − GT‐coated nanoparticles showed faster transit, with a stronger signal in the small intestine than in the stomach. − Bare nanoparticles remained mostly in the ileum's mucus layer. − GT‐NPZ3 had lower intensity in the small intestine compared to GT‐NPZ1 and GT‐NPZ2.	Development of effective oral drug delivery systems	Inchaurraga et al. [69]
	BALB/c mice	Outer membrane vesicles (OMV) obtained from *Escherichia coli/*Gantrez–mannosamine conjugate	− Encapsulation of OMVs with GM‐NPZ delayed gastrointestinal transit compared to free OMVs, as indicated by higher technetium‐99 m levels in the stomach, cecum, and large intestine at 10 h post‐administration. − Free OMVs did not induce specific antibody expression orally, but encapsulation enhanced immunogenicity. − Mice immunized with OMV‐GM‐NPZ showed increased IgG2a levels, confirming a stronger immune response. − Variability in immune responses was observed, with some mice showing higher immunogenicity.	Vaccine development for ETEC infections	Berzosa et al. [74]
	Swiss mice	None/chitosan	− Animals exposed to the highest ZNP‐CS concentration (group ZNP‐CS III) showed an anxiogenic effect, with no signs of sedation or hyperactivity in the EPM test. − ZNP‐CS were able to cross the blood–brain barrier and affected the central nervous system (CNS) of the animals, as indicated by the results in the EPM test. − ZNP‐CS caused depressive‐like behavior, as observed by the increased immobility time in the tail suspension test.	Neurotoxicity and safety	Lima et al. [43]
	Eight‐week‐old BALB/c mice and sows	OMV‐F4 and OMV‐F18/Gantrez AN–Mannosamine Polymer	− A single dose of OMVs (0.1 mg/mouse) from the F4 or F18 *E. coli* strains, either free or encapsulated were used − F4‐GM‐NPZ and F18‐GM‐NPZ presented similar profiles, with significantly higher antibody levels compared to non‐encapsulated antigens. − Nanoencapsulation provided a significant adjuvant effect, enhancing IgA levels in both serum and feces. − The levels of cytokines (IL‐2, IL‐4, IL‐6, TNFα, IFNγ, IL‐10, IL‐17a, and IL‐22) were measured in the splenocytes of mice 28 days after immunization. − Encapsulation of OMVs in nanoparticles led to an increase in IL‐2, IL‐4, and IFN‐γ levels compared to free antigen administration.	Vaccine development for ETEC infection prevention	Matías et al. [42]
	Sprague Dawley (SD) rats	Gambogenic acid/polydopamine	− The blood circulation time of GNA@Zein NPs and GNA@Zein‐PDA NPs increased to 10 h − Cmax of GNA@Zein‐PDA NPs was 2.09 times higher than GNA and 1.29 times higher than GNA@Zein NPs. − *t*_1_/_2_ and MRT of GNA@Zein‐PDA NPs were longer than GNA. − Results indicated that GNA@Zein‐PDA NPs prolonged blood circulation and improved bioavailability.	Cancer treatment and drug delivery research	Zha et al. [34]
		Quercetin/Alginate‐pectin	− Quercetin‐loaded nanoparticles exhibited significantly higher plasma concentrations than quercetin in physical mixtures, with Cmax values of 2.95 mg L^−1^ (ZQ0) and 3.13 mg L^−1^ (ZQ9) compared with 1.33 mg L^−1^ for PM after oral administration. − The half‐life (*t*_1_/_2_) and area under the curve (AUC0–t) were prolonged by nanoparticle encapsulation, reaching 191–224 min and 513–607 mg min L^−1^ for ZQ0 and ZQ9, compared with 136 min and 211 mg min L^−1^ for PM, indicating enhanced systemic exposure. − Calcium reinforcement further improved pharmacokinetics, with ZQ9 showing 6.1% higher Cmax, 17.5% longer *t*_1_/_2_, 18.2% higher AUC, and 9.9% longer mean residence time than ZQ0, suggesting that calcium ions effectively modulate quercetin absorption and bioavailability.	Nutraceutical bioavailability enhancement	Wan et al. [50]
	Albino mice	Cinnamaldehyde and naringin/Lactoferrin and sodium caseinate	− Dual‐coated LF/NaCAS–zein NPs showed enhanced liver targeting with higher fluorescence intensity and lower off‐target accumulation in kidneys and spleen compared to single‐coated NPs or free dye; particle size increased from 209 to 268.5 nm, reducing renal filtration. − CNM/NAR‐loaded NPs alleviated DOX‐induced oxidative stress in liver: GSH increased by 77.6% ± 6.7%, CAT by 56.8% ± 4.9%, and MDA decreased by 63.62% ± 7.3%, whereas free CNM, NAR, or their combination had minimal effect. − Oral administration of CNM/NAR‐loaded NPs preserved body weight and organ weight ratios (liver, kidney, and heart) and restored biochemical markers (ALT, AST, BUN, and creatinine) to near‐normal levels in DOX‐treated mice, unlike free CNM, NAR, or their combination.	Hepatoprotective drug delivery	Mohamed et al. [15]
	Albino rats	Fusidic acid/Lactoferrin	− LF‐ZF‐NPs treatment in rats accelerated wound closure, achieving ~90% closure after 3 weeks, compared to ~84% for Z‐NPs and ZF‐NPs, and ~73% for free FA and LF groups. − Oxidative stress markers improved with LF‐ZF‐NPs: MDA decreased to 212.13 μmol/g protein and SOD increased to 17.51 IU/mL, compared to 385.5 μmol/g protein and 8.24 IU/mL in controls. − Pro‐inflammatory cytokines (TNF‐α, IL‐6, IL‐1β) were significantly regulated after LF‐ZF‐NPs treatment, with early upregulation at day 11 followed by reduction at day 21 versus untreated controls, indicating anti‐inflammatory and healing‐promoting effects.	Wound healing therapy	Marey et al. [64]
	Sprague–Dawley rats and ICR mice	Quercetin/Sodium caseinate and chitosan	− Maximum excretion of quercetin in the suspensions group occurred during 4–6 h, with 38% of the dose, significantly higher than SC‐ZNP (3.9%) and CHC‐ZNP (0.3%). − Accumulative excretion over 24 h: suspensions group (70.0%), SC‐ZNP (19.4%), and CHC‐ZNP (4.6%) with significant differences (*p* < 0.01). − In the stomach, quercetin concentration gradually decreased over time. SC‐ZNP and CHC‐ZNP showed higher retention than the suspension group, though differences were not significant. − In the small intestine, quercetin in SC‐ZNP was significantly higher than in the suspension and CHC‐ZNP groups at 3 and 6 h.	Nanoparticle safety and efficacy in animal models	Zhou et al. [47]
	C57BL/6J mice	Quercetin/Trimethylated chitosan	− TMC‐Zein‐Q nanoparticles achieved the highest plasma quercetin concentration (3.89 ± 1.59 μg/mL) and 2.48‐fold increased relative bioavailability compared with free quercetin (0.61 ± 0.06 μg/mL), outperforming Zein‐Q (2.25 ± 0.37 μg/mL). − TMC‐Zein‐Q significantly reduced body weight in HFD‐fed mice after 1 week (*p* < 0.05) and showed the greatest weight loss after 10 weeks (*p* < 0.01), along with reduced total WAT weight and smaller epididymal adipocytes. − TMC‐Zein‐Q improved lipid metabolism and glucose tolerance, lowering TG, TC, LDL‐c, and increasing HDL‐c; it also enhanced AMPK pathway activity, reversing lipogenesis‐related gene expression more effectively than Zein‐Q or free quercetin.	Oral anti‐obesity delivery	Dai et al. [48]
	Sprague Dawley rats	Atorvastatin/lecithin	− ATV‐Opt‐LCZN‐thermogel significantly reduced knee swelling and nociception in CFA‐induced arthritic rats compared to ATV‐thermogel and CFA groups, with knee swelling at day 28 of 24.67% ± 3.88% versus 37.5% ± 3.56% and nociception scores of 1.58 ± 0.36 versus 3.6 ± 0.45, respectively. − Pro‐inflammatory cytokines (IL‐1β, IL‐6, TNF‐α) were markedly decreased, and anti‐inflammatory cytokines (IL‐4, IL‐10, IL‐13) were increased in the ATV‐Opt‐LCZN‐thermogel group compared to ATV‐thermogel and CFA groups, indicating superior immunomodulatory effects (e.g., IL‐1β: 70.75 ± 5.28 pg/mL vs. 92.48 ± 8.14 pg/mL). − Histopathological analysis revealed minimal cartilage degeneration and mild sub‐synovial inflammation in ATV‐Opt‐LCZN‐thermogel‐treated rats, whereas ATV‐thermogel‐treated rats still showed extensive cartilage and synovial damage, confirming the chondroprotective effect of the nanoparticle formulation.	Osteoarthritis therapy	Elgendy et al. [80]
	ICR mice	Magnolol/chondroitin sulfate	− In comparison to other treatment groups, Mag@CS‐Zein NPsinMPs showed significant therapeutic effects on ulcerative colitis (UC), including alleviated colon shortening, reduced congestion and swelling, and improved survival rate (100% survival in the treatment group). This group also showed better protection against DSS‐induced weight loss and overall improved quality of life for the mice. − Mag@CS‐Zein NPsinMPs significantly increased the expression of ZO‐1 and occludin proteins, which are essential for maintaining intestinal barrier integrity. This indicates that Mag@CS‐Zein NPsinMPs help protect and restore the intestinal barrier in UC	Antioxidant therapy, food preservation	Wang et al. [39]
	ICR mice and AKI mouse	Resveratrol/Hyaluronic acid	− Mice lost weight after CDDP treatment but recovered in the HA‐Zein/Res NPs group. − Kidney organ index and imaging confirmed that HA‐Zein/Res NPs mitigated kidney damage. − CDDP significantly increased BUN and CRE levels, indicating renal injury. − HA‐Zein/Res NPs reduced these levels, suggesting kidney protection.	Reduction of cisplatin‐associated nephrotoxicity	Ning et al. [118]
	H22 tumor‐bearing mice	Celastrol/Hyaluronic acid	− Cel/Zein@HA NPs demonstrated the strongest tumor inhibition among all formulations − Mice treated with free Cel showed significant weight loss, indicating high toxicity. − Cel/Zein NPs and Cel/Zein@HA NPs were well tolerated, showing minimal toxicity. − Tumor sizes in the Cel/Zein@HA NPs group were significantly smaller than in free Cel or Cel/Zein NPs groups. − Immunohistochemistry showed that Cel/Zein@HA NPs significantly reduced: CD31 (6.205‐fold decrease) and VEGF (2.691‐fold decrease), indicating anti‐angiogenic effects; Bcl‐2 expression, which promotes cancer cell survival, was markedly reduced and Bax expression, a pro‐apoptotic protein, was significantly upregulated.	Hepatocellular carcinoma treatment	He et al. [117]
	Sows	OMV‐F4 and OMV‐F18/Gantrez AN–Mannosamine Polymer	− Five weeks after the first immunization, high levels of specific IgG in serum and IgA in feces were noticed, with the highest levels in the NPII vaccinated group. − Two weeks after the second immunization, IgA levels decreased in animals receiving the commercial vaccine, while sows receiving encapsulated vaccines (NPII) had the highest levels. − Offspring piglets from immunized sows (NPI or NPII) had significantly higher levels of IgM, IgA, and IgG antibodies compared to those from non‐immunized mothers.	Vaccine development for ETEC infection prevention	Matías et al. [42]
	Specific pathogen‐free (SPF)‐grade C57BL/6 mice	Curcumin/Shellac	− Zein‐SH‐CurNP treatment effectively alleviated clinical symptoms of DSS‐induced UC, as mice treated with DSS alone lost 34.0% of body weight while Zein‐SH‐CurNP‐treated mice lost only 10.4%. − Zein‐SH‐CurNP improved disease activity index and colon health, reducing DAI values by 8.9% compared with DSS‐treated mice and showing less hematochezia, diarrhea, and colon shortening. − Zein‐SH‐CurNP exhibited strong anti‐inflammatory effects in vivo, markedly decreasing pro‐inflammatory cytokines (TNF‐α, IL‐6, IL‐1β) compared with both DSS‐treated and free curcumin‐treated groups, highlighting the advantages of the nanoparticle delivery system. − GZN nanoparticles significantly reduced blood glucose levels in type 2 diabetic rats over seven weeks compared with saline‐ and Nar‐treated groups, demonstrating improved oral bioavailability and controlled release of Nar. − Insulin tolerance (ITT) and oral glucose tolerance (OGTT) tests confirmed enhanced insulin sensitivity and normalized glucose regulation in GZN NPs‐treated rats. − Histological analysis suggested reparative effects on pancreatic islets and enhanced glycogen storage following nanoparticle treatment. − GZN NPs improved antioxidant capacity, normalizing SOD levels and reducing MDA concentrations in T2D rats. − Liver function and lipid metabolism were also improved, as indicated by reduced ALT, AST, TG, and TC levels, decreased hepatic fat accumulation, and maintenance of normal liver tissue morphology	Colitis‐targeted delivery	Lv et al. [99]
	Sprague–Dawley (SD) rats	Naringin/Glutamine (Gln)‐modified chito‐oligosaccharides		Oral antidiabetic delivery	Guo et al. [40]
Fish	*Oreochromis niloticus*	Eugenol/Chitosan	− The induction time for deep anesthesia was similar across the treatments: eugenol‐80 mg L‐1, NPZMA‐80 mg L‐1, and NPZMA‐40 mg L‐1 (*p* > 0.05). − When eugenol was combined with nanoparticles, NPZMA‐20 mg L‐1 induced significantly faster anesthesia with shorter induction time (*p* < 0.05). − Fish treated with mucoadhesive nanoparticles exhibited reduced responsiveness and immobilization compared to those treated with eugenol‐80 mg L‐1. − The NPZMA‐20 mg L‐1 treatment had the shortest recovery time from anesthesia, while NPZMA‐80 mg L‐1 led to the longest recovery time.	Anesthetic procedures	Ferreira [32]
	*Danio rerio*	Curcumin/dodecamer peptide (G23)‐functionalized polydopamine	− No aggregate formation (> 1 μm) in circulation. − A 49.1% ± 9.2% increase in intravascular fluorescence was observed in Cy3‐ZpD‐G23‐injected larvae compared to Cy3‐NH2, and 36.7% ± 8.7% compared to Cy3‐ZpD. − Cy3‐ZpD‐G23 nanoparticles circulate effectively without aggregation, demonstrating potential for targeted intravenous delivery.	Glioma treatment	Zhang et al. [21, 22, 23]
		Carbamazepine/Chitosan	− CBZ‐NPs significantly reduced toxicity, as pure CBZ caused high mortality (15.56%–32.22% at 50–100 μM) while CBZ‐NPs maintained rates close to the control (~2.2%). − CBZ‐NPs prevented developmental delays and malformations, since pure CBZ markedly decreased larval emergence (74.44% and 57.78%) and increased malformations (13.13%–21.35%), whereas CBZ‐NPs kept emergence above 95% with far fewer malformations. − CBZ‐NPs improved neurological responses in epileptic larvae, showing higher frequencies of head and tail reflexes and reduced expression of neurotoxicity markers (5HT4 and BMAL1) compared with pure CBZ.	Epilepsy therapy	Alak et al. [79]
Worm	*Caenorhabditis elegans*	Insulin/GantrezAN‐thiamine conjugate	− Nanoencapsulated insulin significantly reduced fat accumulation in nematodes under high glucose conditions (*p* < 0.001). − I‐GT‐NP2 achieved the greatest reduction (22%), outperforming free insulin. − Encapsulation enhanced insulin efficacy, with I‐GT‐NP1 reducing fat by 15% and I‐NP by 10%, demonstrating superior metabolic regulation.	Drug delivery/antidiabetic treatments	Inchaurraga et al. [70]
		Insulin/Gantrez AN‐PEG conjugate	− Nanoencapsulated insulin (I‐ZNP‐GP) induced a 33% reduction in fat accumulation in wild‐type N2 nematodes, significantly outperforming free insulin (14%) and I‐ZNP (22%) (*p* < 0.001). − The lower glucose content in worms treated with nanoencapsulated insulin (40% reduction, *p* < 0.01) suggests better glucose regulation compared to free insulin (32%, *p* < 0.05).	Drug delivery/antidiabetic treatments	Martínez‐López et al. [71]
		Insulin/PEG	− Free insulin (50 μg/mL) induced a 15% reduction in fat accumulation in *C. elegans* compared to controls (*p* < 0.01). − I‐NP‐treated worms (insulin‐loaded zein nanoparticles) showed a 30% decrease in fat accumulation compared to control worms. − I‐NP‐PEG (insulin‐loaded nanoparticles with PEG coating) induced a 40% reduction in fat, approaching the effect of Orlistat (42%). − Empty zein nanoparticles, both bare and PEG‐coated, also led to a fat reduction of about 11% and 14%, respectively	Drug delivery/antidiabetic treatments	Reboredo et al. [72]

#### 5.2.1 Mammals

Studies on ZNPs in rats and mice are essential for assessing safety, efficacy, and therapeutic potential, leveraging their genetic and physiological similarities to humans [109]. Studies on systemic toxicity, dose–response, and tolerance help predict ZNPs' risks and benefits, supporting their role in translational research for diseases like diabetes, obesity, cardiovascular diseases, and cancer [121].

Our study revealed that most in vivo studies on ZNPs have been conducted in mammals. Among these studies, the primary focus has been on their anticancer properties, with a particularly noteworthy finding being their reduced toxicity [117, 118]. Another promising area of research is their role in enhancing drug bioavailability, particularly as drug delivery systems, as seen with insulin [61, 73]. Additionally, ZNPs have shown great potential as platforms for developing more efficient vaccines [42, 74].

#### 5.2.2 Fish

Research on nanoparticles in fish is vital for assessing environmental impact, human health risks, and ecological concerns like bioaccumulation and biomagnification [122]. *Oreochromis niloticus* is an important in vivo model for studying nanoparticle‐based anesthesia, toxicology, environmental impact, and tissue regeneration, aiding drug and therapy development [123]. Adittionaly, zebrafish ( *Danio rerio* ) serves as an in vivo model for toxicity and genetic studies, offering rapid development, high genetic similarity to humans, and transparent embryos for easy organ observation [124, 125].

#### 5.2.3 Nematodes

Studies on the biological activity and toxicology of coated ZNPs using the in vivo model *Caenorhabditis elegans* ( *C. elegans* ) are essential for understanding their efficacy and safety in therapeutic and industrial applications. *C. elegans* is a widely used nematode model in life and environmental sciences due to its sensitivity to pollutants, short life cycle (3.5 days), self‐fertilization, and high reproductive capacity (300 eggs per worm) [126]. Its genetic similarity to humans is approximately 41.7%, ranging from 20% to 71% [127]. This model is valuable for transgenerational and multigenerational toxicity studies, assessing endpoints such as behavior, lifespan, survival, reproduction, biochemical changes (e.g., oxidative stress, enzymatic activity), gene expression, and larval development [128].

## 6 Conclusion

In summary, coated zein nanoparticles represent a versatile and biocompatible platform for drug delivery, combining prolonged release, low toxicity, and broad applicability. This review addressed a gap in the literature by summarizing synthesis methods, coating strategies, and in vitro and in vivo applications, offering a practical reference for the design and screening of new systems with potential clinical translation. Evidence shows that coated ZNPs outperform uncoated particles in stability, targeted action, and biological response. Further research should explore optimized coating approaches, long‐term safety, and more robust in vivo models to support their advancement toward clinical applications.

## Author Contributions

Conceptualization: M.F. Lima. Methodology: M.F. Lima. Investigation: M.F. Lima. Formal analysis: M.F. Lima. Data curation: M.F. Lima and A.J.T.H. Huisa. Writing – original draft: M.F. Lima. Writing – review and editing: M.F. Lima, A.J.T.H. Huisa, I.D.L. Cavalcanti, K.B. Araújo, C.B.G. Silva, A.F. Silva Neto, M.C. Britto Lira‐Nogueira, N.S.S. Magalhães, and P. Gubert. Visualization: M.F. Lima, A.F. Silva Neto, and C.B.G. Silva. Supervision: N.S.S. Magalhães and P. Gubert. Project administration: P. Gubert. Funding acquisition: N.S.S. Magalhães.

## Funding

This work was supported by the Foundation Coordination for the Improvement of Higher Education Personnel (CAPES) process number: 88887.695461/2022‐00. We would like to thank the FACEPE ‐ Fundação de Amparo à Ciência e Tecnologia de Pernambuco (APQ‐1223‐2.05/22), the National Council for Scientific and Technological Development (CNPq) for financial support of the National Institute of Science and Technology on Molecular Science (INCT‐CiMol ‐ 406804/2022‐2) and the Keizo Asami Institute (iLIKA) for providing research infrastructure.

## Ethics Statement

The authors have nothing to report.

## Consent

All the authors have read the manuscript and have approved this submission.

## Conflicts of Interest

The authors declare no conflicts of interest.

## Data Availability

All data generated or analyzed during this study are included in this published article.
